# Supplementary material for: Bacillus subtilis Spore Resistance to Simulated Mars Surface Conditions
Source: Front Microbiol. 2019 Feb 26;10:333. doi: 10.3389/fmicb.2019.00333 (PMC6399134; doi:10.3389/fmicb.2019.00333)
Supplement: Supplementary file 1 [file Data_Sheet_1.PDF]

frontiers

Supplementary Material

Bacillus subtilis spore resistance to simulated Mars surface conditions

Marta Cortesão[1], Felix M. Fuchs[1], Fabian M. Commichau[2], Patrick Eichenberger[3], Andrew C. Schuerger[4], Wayne L. Nicholson[5], Peter Setlow[6], and Ralf Moeller[1,*]

*** Correspondence:** German Aerospace Center (DLR e.V.), Institute of Aerospace Medicine, Radiation Biology Department, Space Microbiology Research Group, Linder Höhe, D-51147 Cologne (Köln), Germany, Phone +49(2203) 601-3145, Fax +49(2203) 61790, E-mail: ralf.moeller@dlr.de

1 Supplementary Figures

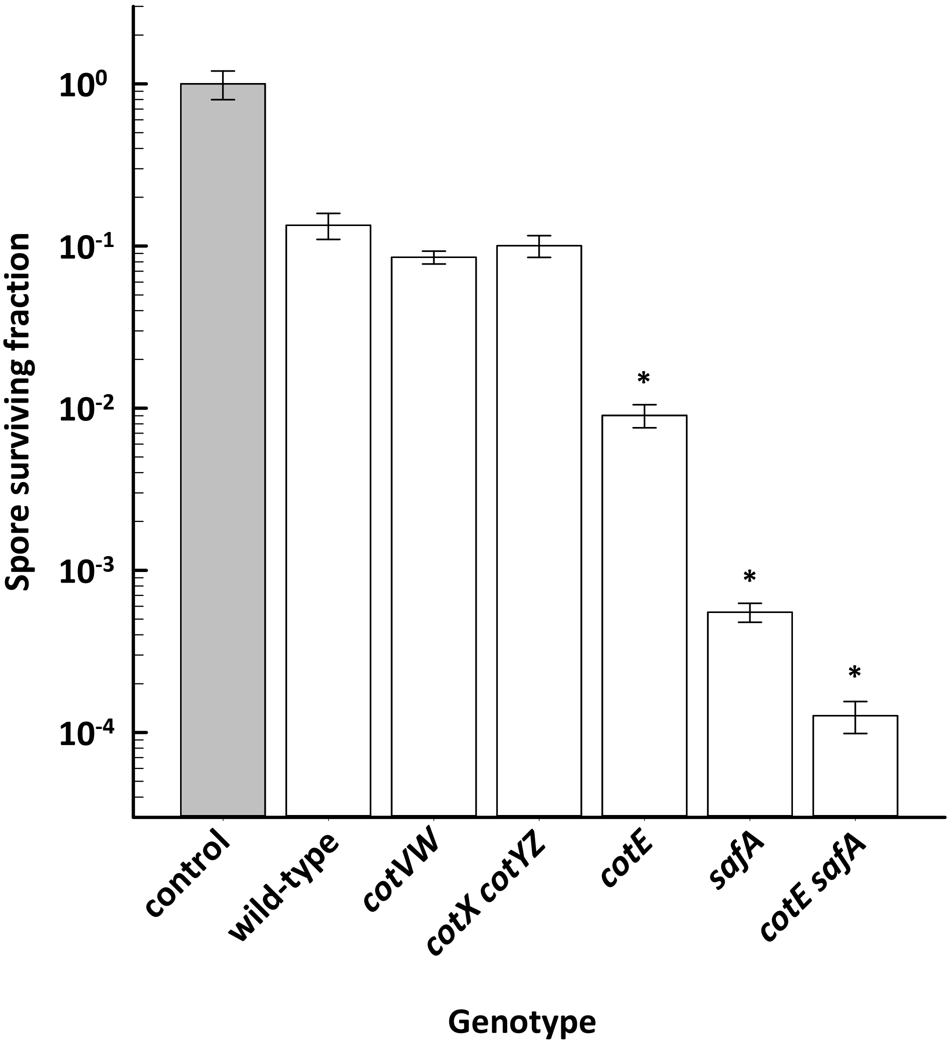

Supplementary Figure S01. Survival of *B. subtilis* coat-defective spores exposed to M(+)UV. Spores were exposed to M(+)UV and spore viability was determined as described in Methods. The genotypes tested are indicated below the bars; the wild-type strain is PY79. The control spores of each strain were air-dried spore multilayers exposed for 24 h to ambient laboratory conditions, and all control spore surviving fractions were set at 100%. Data are expressed as averages and standard deviations (n = 3). (*) Statistically significant difference from values for wild-type spores ($P \leq 0.05$).

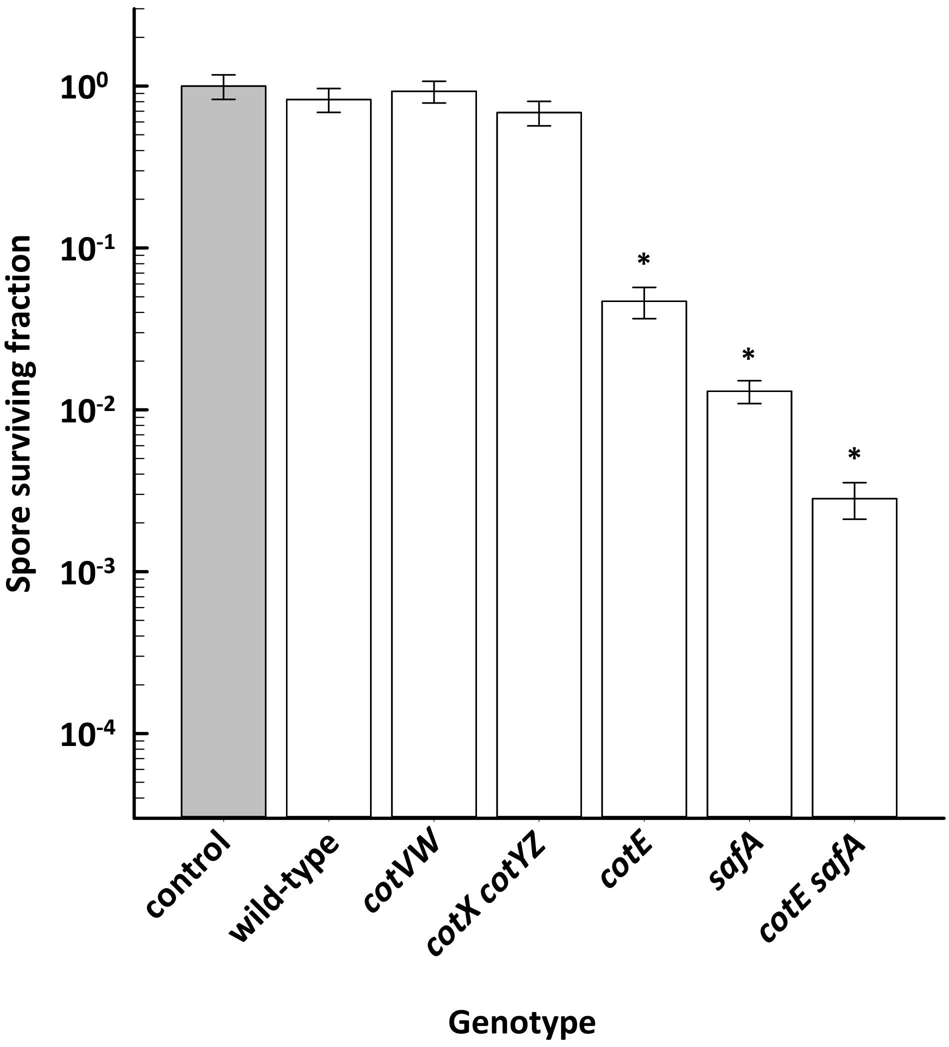

Supplementary Figure S02. Survival of *B. subtilis* coat-defective spores exposed to M(-)UV. Spores were exposed to M(-)UV and spore viability was determined as described in Methods. The genotypes tested are indicated below the bars; the wild-type strain is PY79. The control spores of each strain were air-dried spore multilayers exposed for 24 h to ambient laboratory conditions, and all control spore surviving fractions were set at 100%. Data are expressed as averages and standard deviations (n = 3). (*) Statistically significant difference from values for wild-type spores ($P \leq 0.05$).

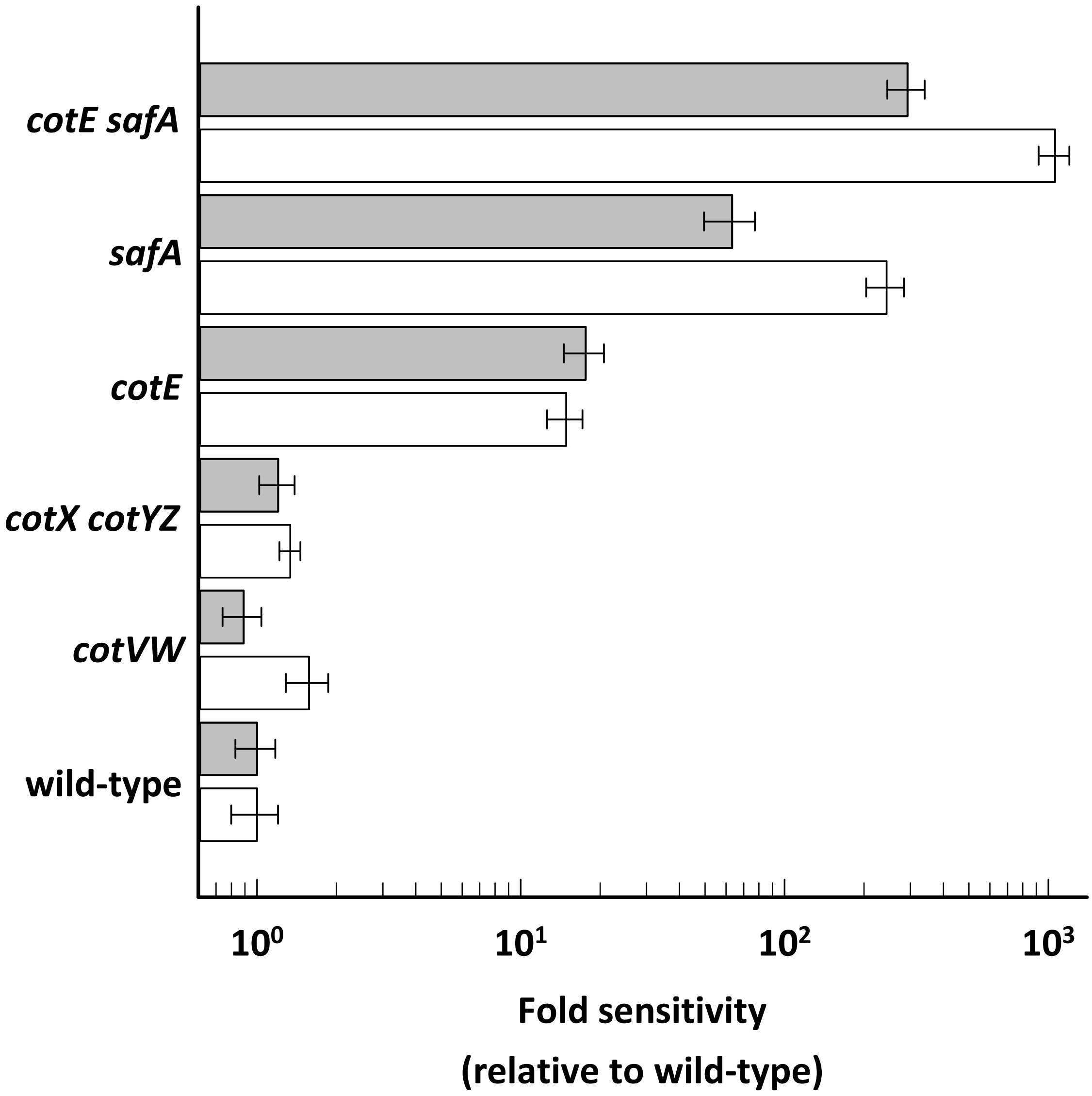

Supplementary Figure S03. Cortesão et al. Impact of coat defects on relative sensitivities of spores to M(+)UV (grey) and M(-)UV (white). Relative spore sensitivity was expressed as the ratio of the survival of wild-type spores to that of mutant spores using data from Figure S01 and S02. Data are averages and standard deviations (n = 3). The actual data are shown above the corresponding columns in Table 4.

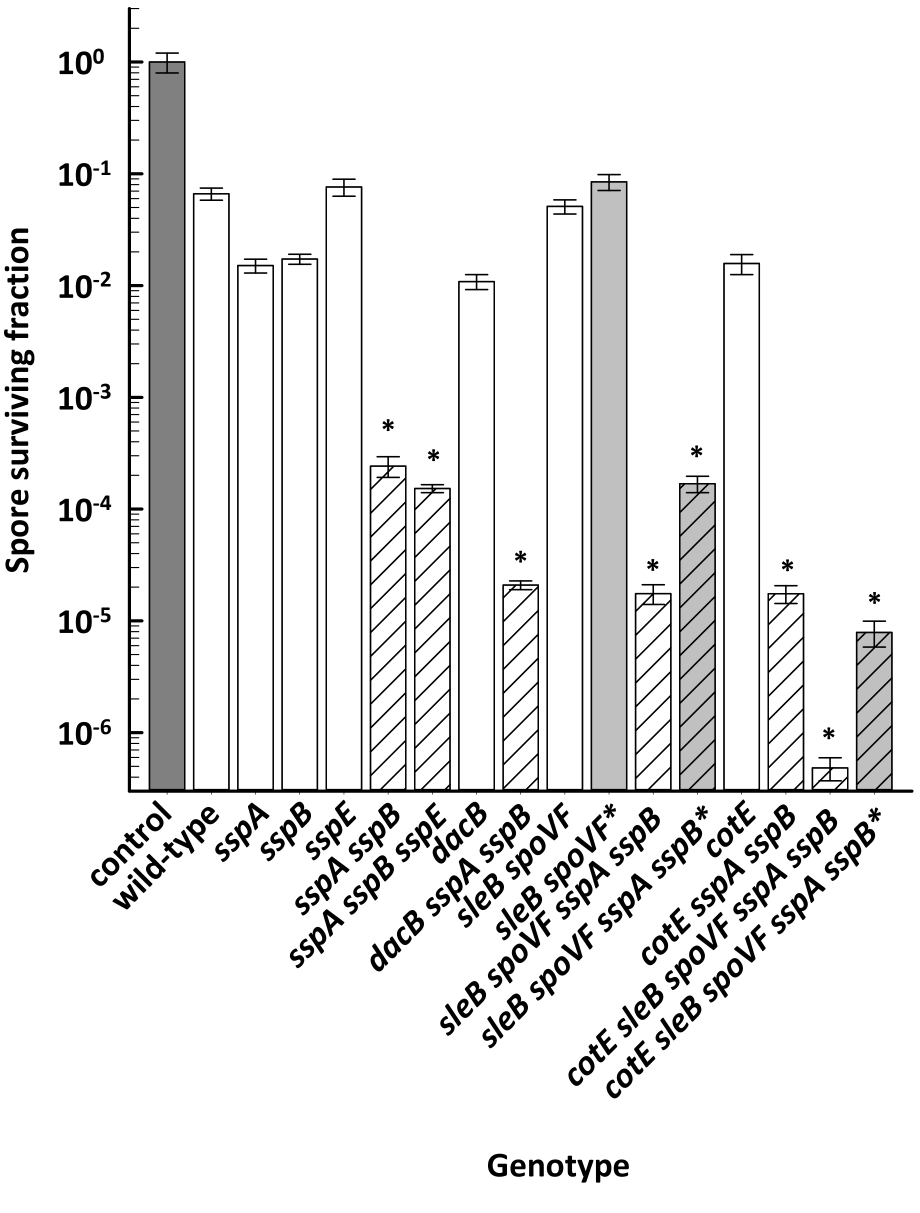

Supplementary Figure S04. Cortesão et al. Survival of *B. subtilis* spores lacking protective components exposed to M(+)UV. Spores were exposed to M(+)UV and spore viability was determined as described in Methods. The strains used are indicated below the bars (Table 1); the wild-type strain is PS832. The control spores of each strain were air-dried spore multilayers exposed for 24 h to ambient laboratory conditions, and all control spore surviving fractions were set at 100%. Data are expressed as averages and standard deviations (n = 3). Dashed bars: mutants with α- and β-type SASP deficiency. In (light) grey: 100 µM Ca^{2+} and DPA added during sporulation. (*) Statistically significant difference from values for wild-type spores ($P \leq 0.05$).

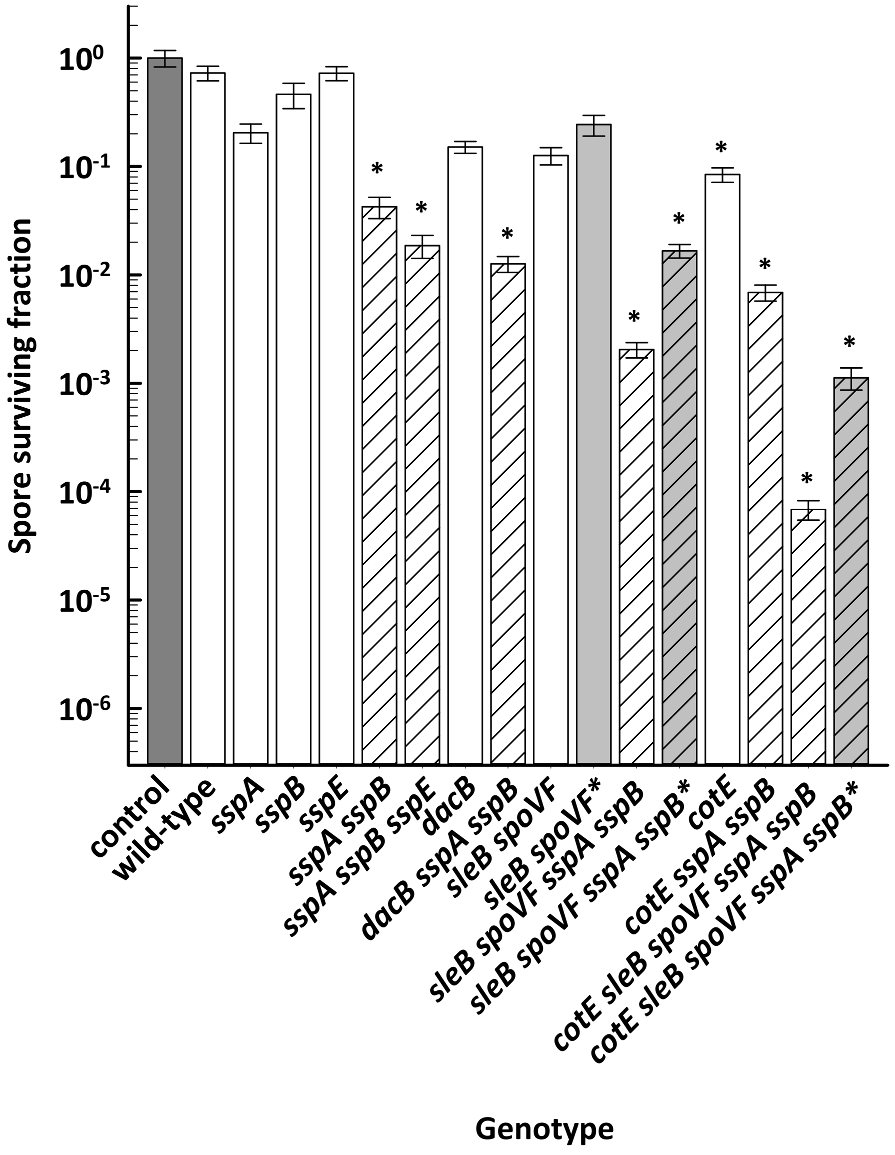

Supplementary Figure S05. Cortesão et al. Survival of *B. subtilis* spores lacking protective components exposed to M(-)UV. Spores were exposed to M(-)UV and spore viability was determined as described in Methods. The strains used are indicated below the bars (Table 1); the wild-type strain is PS832. The control spores of each strain were air-dried spore multilayers exposed for 24 h to ambient laboratory conditions, and all control spore surviving fractions were set at 100%. Data are expressed as averages and standard deviations (n = 3). Dashed bars represent mutants with α- and β-type SASP deficiency. In (light) grey: 100 µM Ca^{2+} and DPA added during sporulation. (*) Statistically significant difference from values for wild-type spores ($P \leq 0.05$).

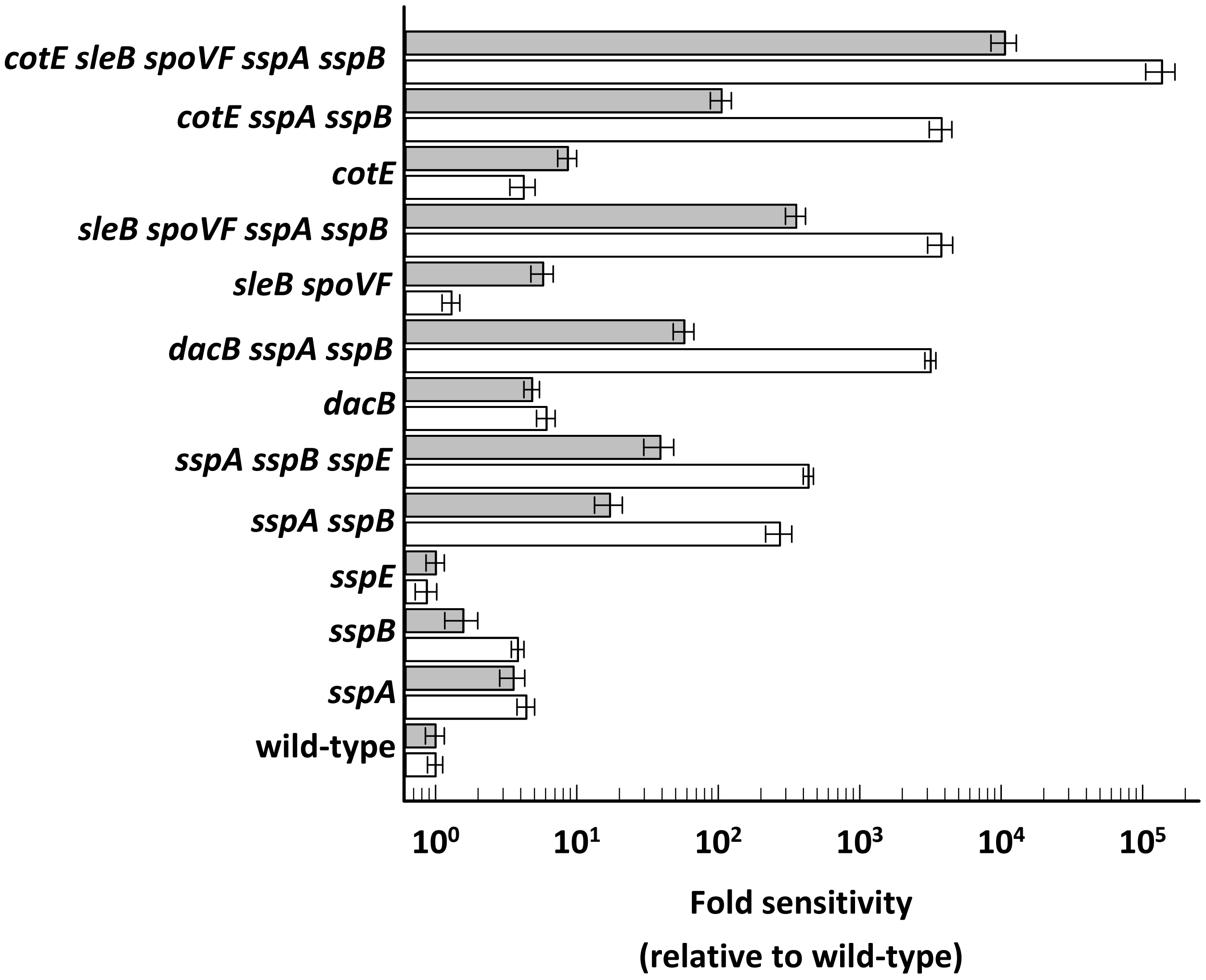

Supplementary Figure S06. Cortesão et al. Impacts of loss of various spore protective factors on relative sensitivities of spores to M(+)UV (grey) and M(-)UV (white). Relative spore sensitivity was expressed as the ratio of the survival of wild-type spores to that of mutant spores using data from Figure S04 and S05. Data are averages and standard deviations (n = 3). The actual data are shown above the corresponding columns in Table 4.

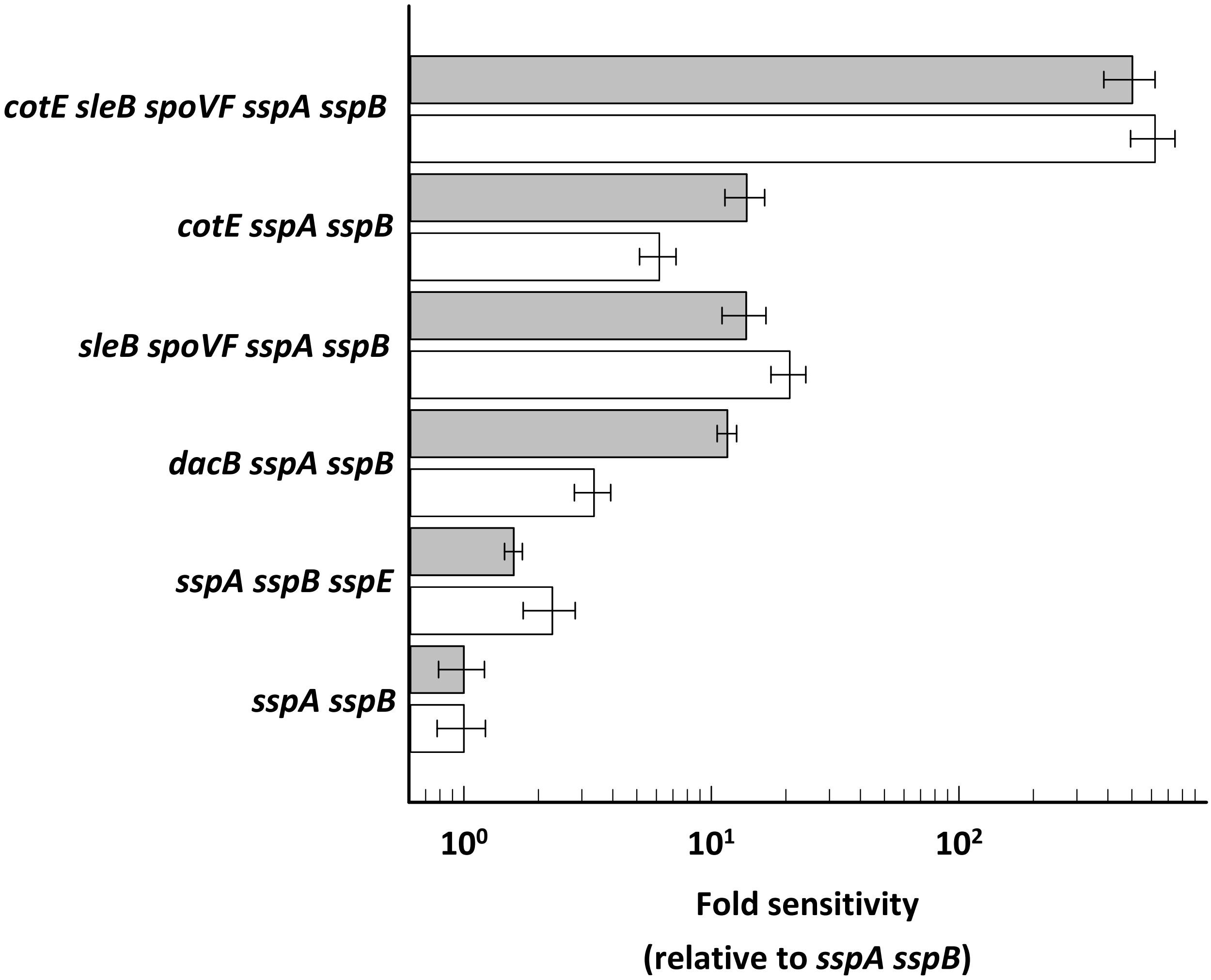

Supplementary Figure S7. Cortesão et al. Impacts of loss of *sspA sspB* genes on relative sensitivities of other mutant spores to M(+)UV (grey) and M(-)UV (white). Relative spore sensitivity was expressed as the ratio of the survival of *sspA sspB* individual mutant spores to that of spores with additional mutations using data from Figure S04 and S05. Data are averages and standard deviations (n = 3). The actual data are shown above the corresponding columns in Table 4.

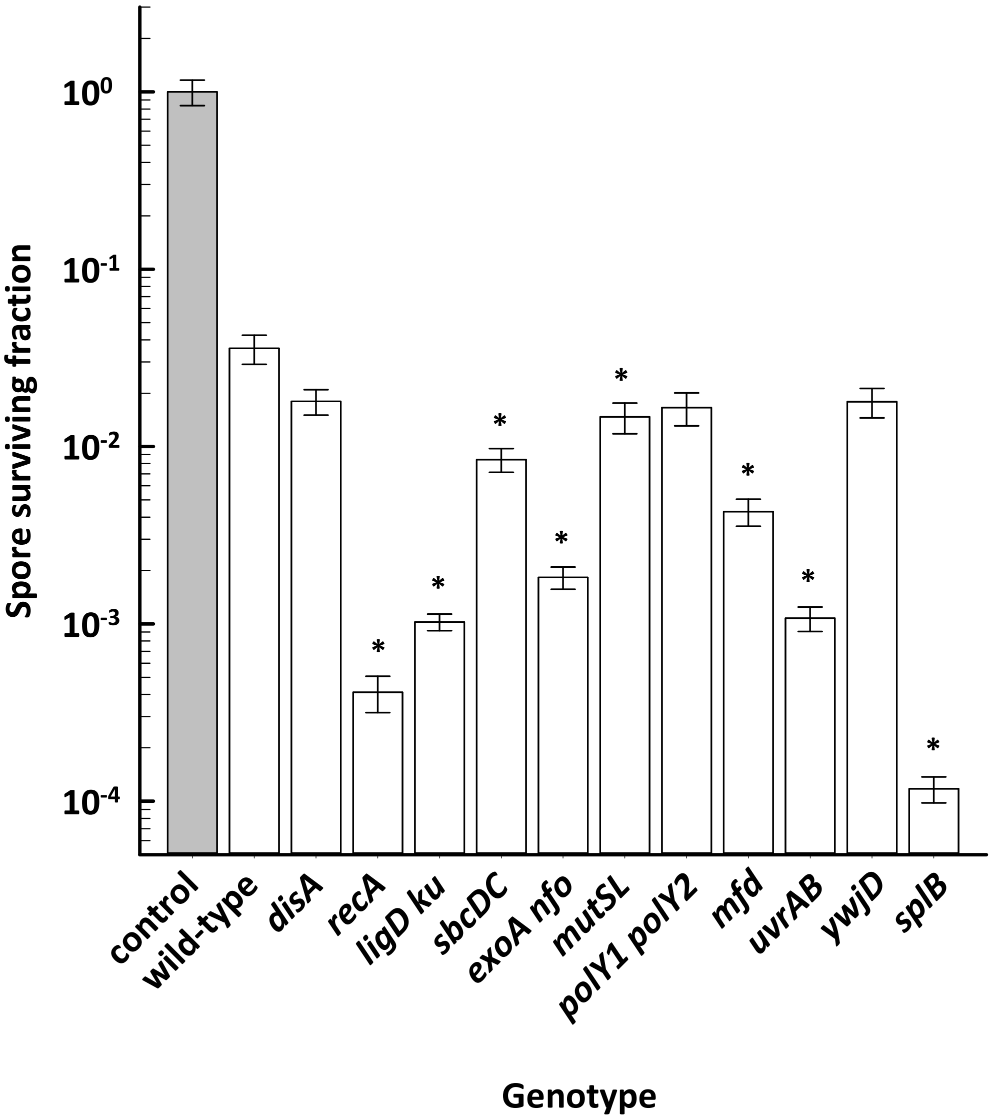

Supplementary Figure S08. Cortesão et al. Survival of *B. subtilis* spores lacking various DNA repair genes exposed to M(+)UV. Spores were exposed to M(+)UV and spore viability was determined as described in Methods. The strains used are indicated below the bars (Table 2); the wild-type strain is 168. The control spores of each strain were air-dried spore multilayers exposed for 24 h to ambient laboratory conditions, and all control spore surviving fractions were set at 100%. Data are expressed as averages and standard deviations (n = 3). (*) Statistically significant difference from values for wild-type spores ($P \leq 0.05$).

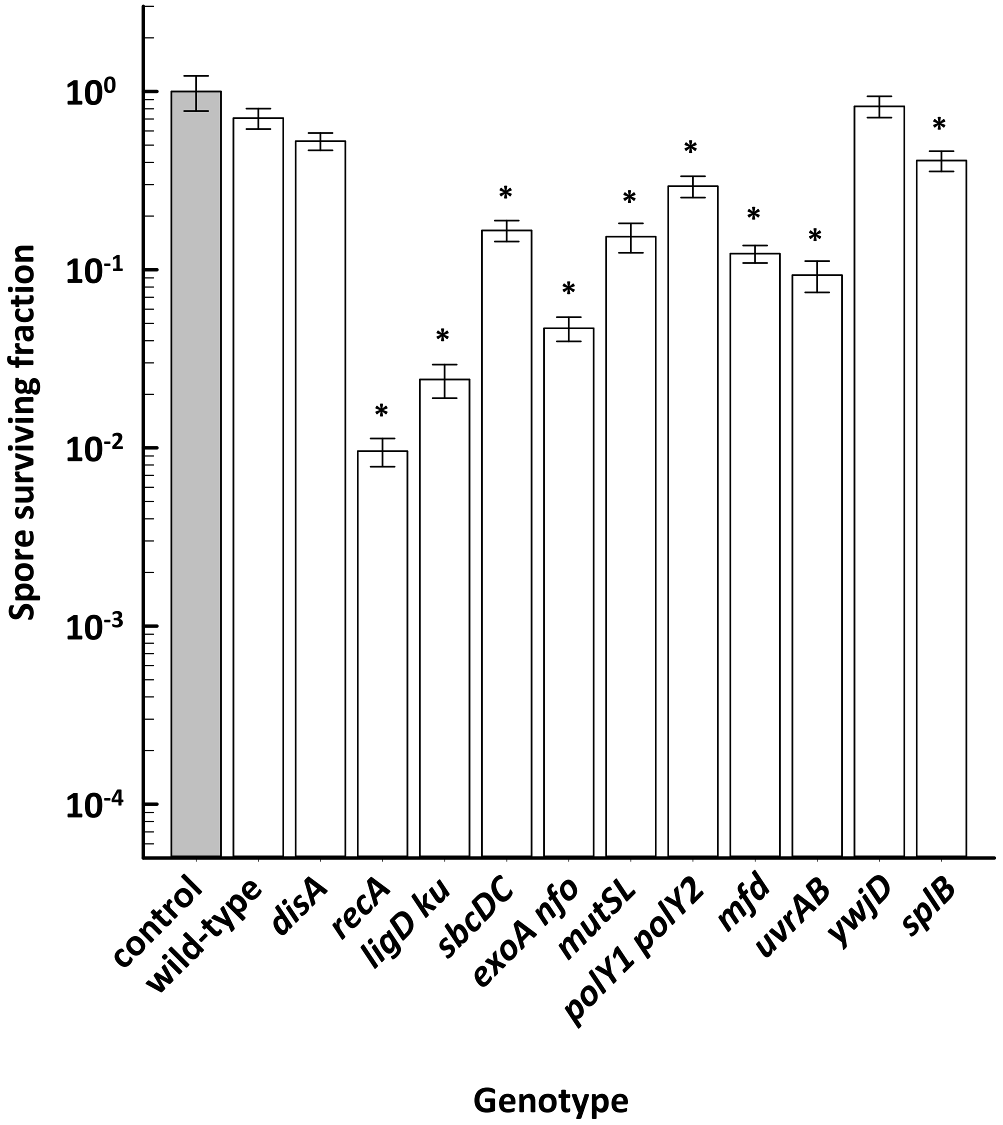

Supplementary Figure S09. Cortesão et al. Survival of *B. subtilis* spores lacking various DNA repair genes exposed to M(-)UV. Spores were exposed to M(-)UV and spore viability was determined as described in Methods. The strains used are indicated below the bars (Table 2); the wild-type strain is 168. The control spores of each strain were air-dried spore multilayers exposed for 24 h to ambient laboratory conditions, and all control spore surviving fractions were set at 100%. Data are expressed as averages and standard deviations (n = 3). (*) Statistically significant difference from values for wild-type spores ($P \leq 0.05$).

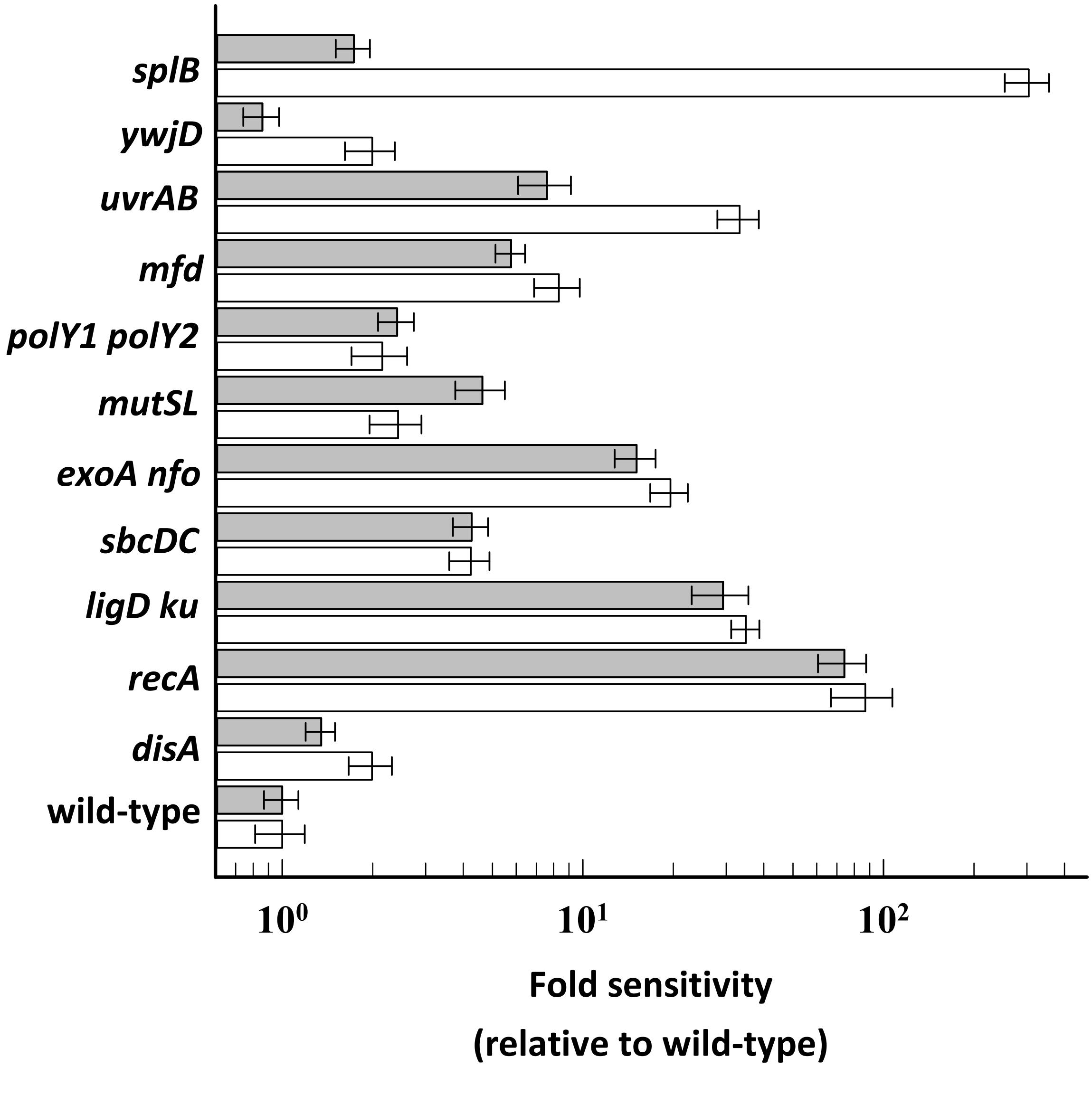

Supplementary Figure S10. Cortesão et al. Impact of loss of various DNA repair genes on relative sensitivities of spores to M(+)UV (grey) and M(-)UV (white). Relative spore sensitivity was expressed as the ratio of the survival of wild-type spores (the wild-type strain is 168) to that of mutant spores using data from Figure S08 and S09. Data are averages and standard deviations (n = 3). The actual data are shown above the corresponding columns in Table 5.

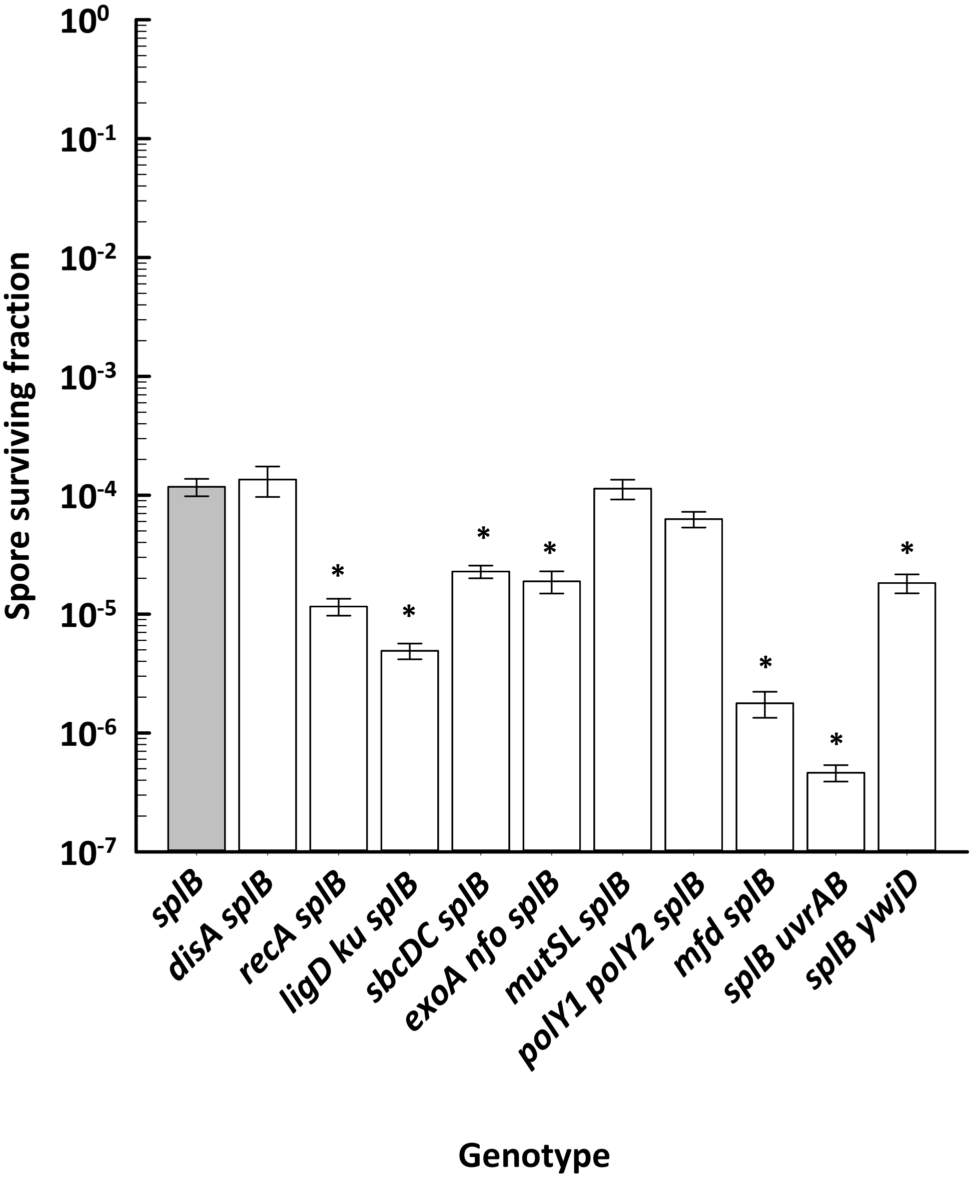

Supplementary Figure S11. Cortesão et al. Survival of *B. subtilis* spores lacking the *splB* gene together with other DNA repair genes exposed to M(+)UV. Spores were exposed to M(+)UV and spore viability was expressed relative to that of the *splB* strain and was determined as described in Methods. The strains used are indicated below the bars. The control spores of each strain were air-dried spore multilayers exposed for 24 h to ambient laboratory conditions, and all control spore surviving fractions were set at 100%. Data are expressed as averages and standard deviations (n = 3). (*) Statistically significant difference from values for wild-type spores ($P \leq 0.05$).

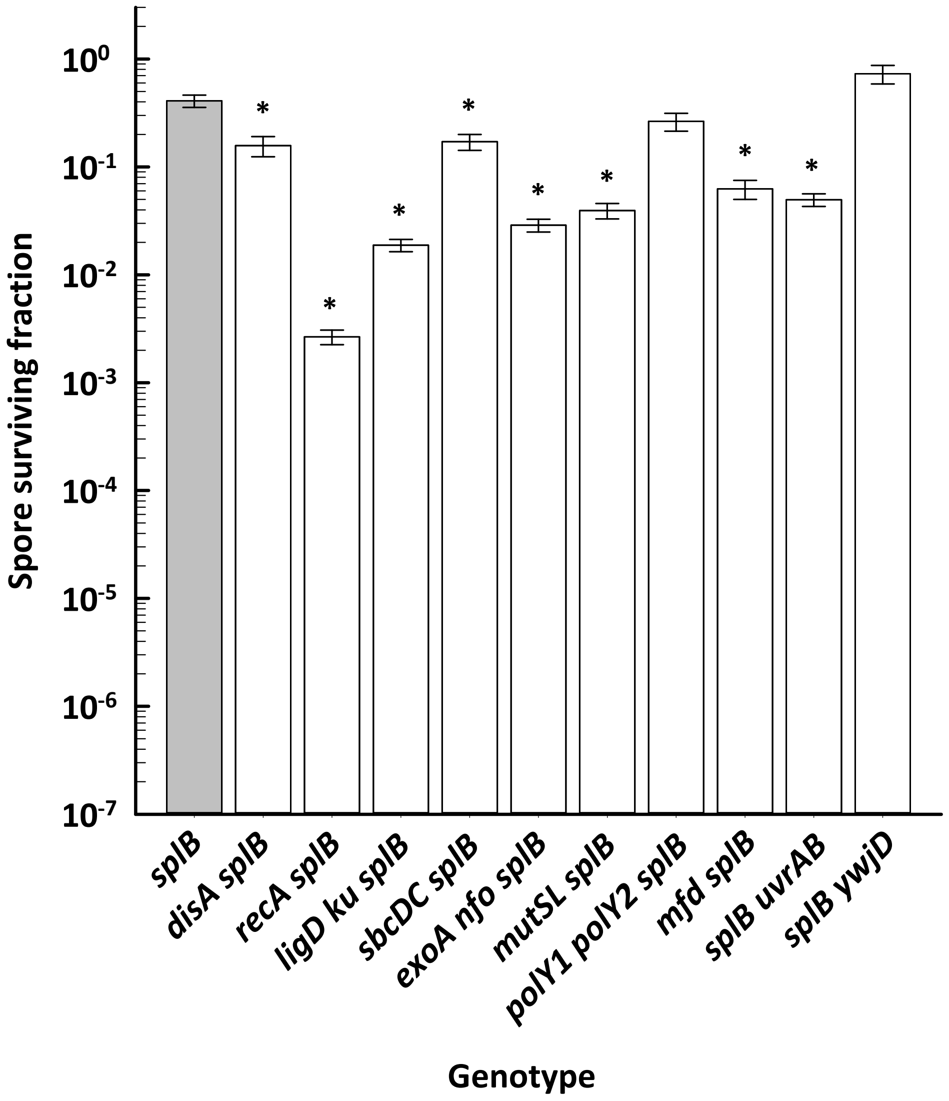

Supplementary Figure S12. Cortesão et al. Survival of *B. subtilis* spores lacking the *splB* gene together with other DNA repair genes exposed to M(+)UV. Spores were exposed to M(+)UV and spore viability was expressed relative to that of the *splB* strain and was determined as described in Methods. The strains used are indicated below the bars. The control spores of each strain were air-dried spore multilayers exposed for 24 h to ambient laboratory conditions, and all control spore surviving fractions were set at 100%. Data are expressed as averages and standard deviations (n = 3). (*) Statistically significant difference from values for wild-type spores ($P \leq 0.05$).

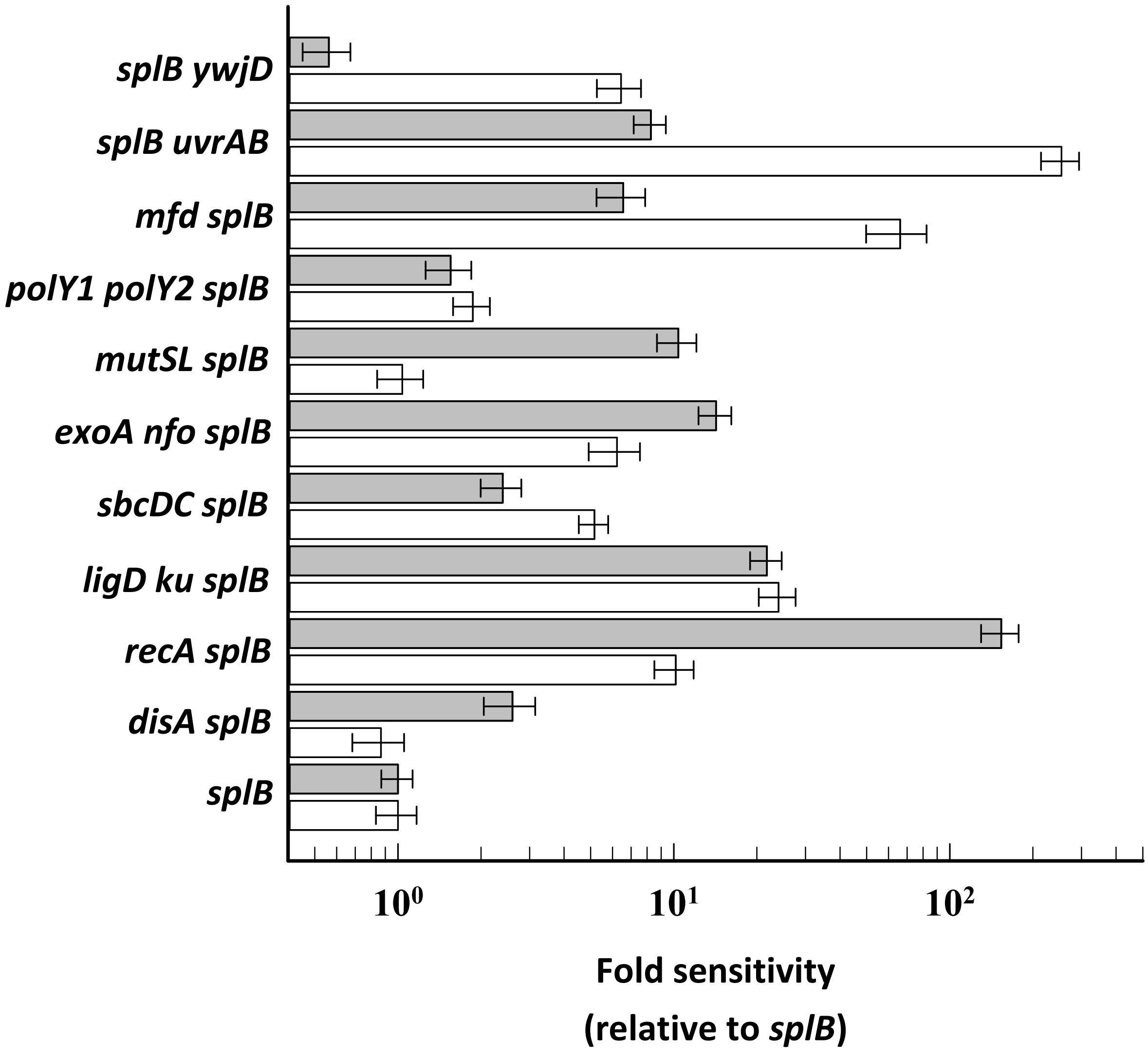

Supplementary Figure S13. Cortesão et al. Impact of loss of the *splB* gene on relative sensitivities of other mutant spores to M(+)UV (grey) and M(-)UV (white). Relative spore sensitivity was expressed as the ratio of the survival of *splB* spores to that of double mutant spores using data from Figure S11 and S12. Data are averages and standard deviations (n = 3). The actual data are shown above the corresponding columns in Table 5.
