# Supplementary material for: Bacillus subtilis Spore Resistance to Simulated Mars Surface Conditions
Source: Front Microbiol. 2019 Feb 26;10:333. doi: 10.3389/fmicb.2019.00333 (PMC6399134; doi:10.3389/fmicb.2019.00333)
Supplement: Supplementary file 2 [file Data_Sheet_2.ZIP › revised manuscript.pdf]

Bacillus subtilis spore resistance to simulated Mars surface conditions

Marta Cortesão[1], Felix M. Fuchs[1], Fabian M. Commichau[2], Patrick Eichenberger[3], Andrew C. Schuerger[4], Wayne L. Nicholson[5], Peter Setlow[6], and Ralf Moeller[1,*]

[1]German Aerospace Center (DLR), Institute of Aerospace Medicine, Radiation Biology Department, Space Microbiology Research Group, Cologne (Köln), Germany

[2]University of Göttingen, Institute for Microbiology and Genetics, Department of General Microbiology, Göttingen, Germany

[3]New York University, Center for Genomics and Systems Biology, Department of Biology, New York, New York, USA

[4]University of Florida, Department of Plant Pathology, Space Life Sciences Laboratory, Merritt Island, Florida, USA

[5]University of Florida, Department of Microbiology and Cell Science, Space Life Sciences Laboratory, Merritt Island, Florida, USA

[6]University of Connecticut Health Center (UConn Health), Department of Molecular Biology and Biophysics, Farmington, Connecticut, USA

*** Correspondence:** German Aerospace Center (DLR e.V.), Institute of Aerospace Medicine, Radiation Biology Department, Space Microbiology Research Group, Linder Höhe, D-51147 Cologne (Köln), Germany, Phone +49(2203) 601-3145, Fax +49(2203) 61790, E-mail: ralf.moeller@dlr.de

Keywords: *Bacillus subtilis*[1], spore resistance[2], DNA repair[3], SASP[4], Mars[5], contamination[6], radiation[7], planetary protection[8]

Abstract

In a Mars exploration scenario, knowing if and how highly resistant *Bacillus subtilis* spores would survive on the Martian surface is crucial to design planetary protection measures and avoid false positives in life-detection experiments. Therefore, in this study a systematic screening was performed to determine whether *B. subtilis* spores could survive an average day on Mars. For that, spores from two comprehensive sets of isogenic *B. subtilis* mutant strains, defective in DNA protection or repair genes, were exposed to 24 h of simulated Martian atmospheric environment with or without 8 h of Martian UV radiation [M(+)UV and M(-)UV, respectively]. When exposed to M(+)UV, spore survival was dependent on: (1) core dehydration maintenance, (2) protection of DNA by α/β-type small acid soluble proteins (SASP), and (3) removal and repair of the major UV photoproduct (SP) in spore DNA. In turn, when exposed to M(-)UV, spore survival was mainly dependent on protection by the multilayered spore coat, and DNA double-strand breaks represent the main lesion accumulated. *Bacillus subtilis* spores were able to survive for at least a limited time in a simulated Martian environment, both with or without solar UV radiation. Moreover, M(-)UV-treated spores exhibited survival rates significantly higher than the M(+)UV-treated spores. This suggests that on a real Martian surface, radiation shielding of spores (e.g., by dust, rocks, or spacecraft surface irregularities) might

significantly extend survival rates. Mutagenesis were strongly dependent on the functionality of all structural components with small acid-soluble spore proteins, coat layers and dipicolinic acid as key protectants and efficiency DNA damage removal by AP endonucleases (ExoA and Nfo), non-homologous end joining (NHEJ), mismatch repair (MMR) and error-prone translesion synthesis (TLS). Thus, future efforts should focus on: (1) determining the DNA damage in wild-type spores exposed to M(+/-)UV and (2) assessing spore survival and viability with shielding of spores via Mars regolith and other relevant materials.

1 Introduction

Mars is a cold and dry planet, with intense UV (190-400 nm) and ionizing radiation in the form of galactic cosmic radiation (GCR) and solar particle events (SPE) (Guo *et al.*, 2018). The Martian atmosphere is also highly oxidizing due to the OH radicals and oxygen atoms produced by photolysis which result in surface oxidation and the formation of O_2, O_3 and H_2O_2 (Gargaud *et al.*, 2011). In addition, the Mars surface exhibits: temperature shifts from -125 °C to +20 °C; extremely low water vapor pressure (Davila *et al.*, 2010; Fox-Powell *et al.*, 2016); and very low atmospheric pressure. These extreme conditions are stressful to all known life forms, causing physiological, biochemical and structural damage, which can be lethal for most terrestrial organisms (Jakosky *et al.*, 2003). At the molecular level, this damage can affect membrane lipids, proteins, RNA and, most importantly, DNA. Specific DNA damage includes single strand breaks (SSB), double strand breaks (DSB), and photolesions such as cyclobutane-type pyrimidine dimers (CPDs), 6-4 photoproducts (6-4 PPs) and the thymine dimer 5-thyminyl-5,6-dihydrothymine, commonly known as the spore photoproduct (SP) (Setlow, 2014).

Nonetheless, despite complex stress-induced damage, spores of the Gram-positive bacterium *Bacillus subtilis* have repeatedly demonstrated their resistance to many space-related extremes, becoming one of the model organisms in the field of Space Microbiology. Studies have shown *Bacillus* spores survive in extreme dryness, high levels of UV and ionizing radiation, and outer space conditions in Low Earth Orbit (LEO), where they were exposed to solar UV, high vacuum, GCR, and temperature fluctuations (Dose *et al.*, 1995; Horneck *et al.*, 2001; Nicholson and Schuerger, 2005; Fajardo-Cavazos *et al.*, 2010; Horneck *et al.*, 2010; Moeller *et al.*, 2012b).

Because of their extreme resistance, spores of *B. subtilis,* and other spore-forming bacteria, present a challenge for bio-sterilization in spacecraft facilities, calling for the development of new and more efficient sterilization regimens (Stapelmann *et al.*, 2013; Khodadad *et al.*, 2017). *Bacillus subtilis* spores were also shown to survive in Mars analogue soils, confirming a potential forward contamination risk to Mars sites with liquid brines (Schuerger *et al.*, 2017).

Resistance of spores to extreme conditions does not rely on one single mechanism, but rather on a combination of several strategies (Setlow, 2014). The first line of action is “damage prevention”. The overall spore structure is composed of the core, inner membrane, cortex, coat, and crust layers (Figure 1), and has a wide number of properties and components that protect spores from many stress factors. Specifically, the spore core has low water content (25 to 55 % of wet weight), due in some fashion to the spore’s peptidoglycan cortex, that provides resistance to wet heat. Within the core, high levels (~25% of core dry weight) of pyridine-2,6-dicarboxylic acid - dipicolinic acid (DPA), in a 1:1 chelate with Ca^{2+} (Ca-DPA) help to protect spores from desiccation and DNA-damaging agents and maintain

spore dormancy (Magge *et al.*, 2008). The core's high levels of α/β-type small, acid-soluble spore proteins (SASP) (Magge *et al.*, 2008) that saturate spore DNA are one of the main factors protecting spores from genotoxic chemicals, desiccation, dry and wet heat, as well as UV and γ-radiation (Mason and Setlow, 1986; Moeller *et al.*, 2008). Moreover, the thick proteinaceous coat and crust layers, as well as the inner membrane, function as barriers to many toxic chemicals minimizing their ability to access the spore core where DNA and most spore enzymes are located. The spore coats also contain melanin-like pigments that absorb UV radiation, and there is evidence that such pigments can play a significant role in spore resistance to UV-B and UV-A radiation (Hullo et al., 2001; Moeller et al., 2008; Moeller et al., 2014; Setlow, 2014).

The second line of defense is "damage repair", which takes place soon after spores germinate and begin outgrowth. *Bacillus subtilis* spores are armed with enzymes of multiple DNA repair pathways, thus marshalling multiple mechanisms that ensure spore survival. The main known mechanisms for repair of DNA damage in spores are: (1) homologous recombination (HR), (2) non-homologous end joining (NHEJ), (3) nucleotide excision repair (NER), (4) DNA integrity scanning, (4) inter-strand cross-link repair, (5) base excision repair (BER), (6) SP repair by spore photoproduct lyase (Spl), (7) mismatch repair (MMR), (8) endonuclease-dependent excision repair (UVER), and (9) error-prone translesion synthesis (TLS) (Xue and Nicholson, 1996; Rebeil *et al.*, 1998; Duigou *et al.*, 2005; Moeller *et al.*, 2007c; Lenhart *et al.*, 2012; Moeller *et al.*, 2012a).

The continuous and ongoing efforts to characterize the geochemistry, mineralogy and consequent habitability of the Martian surface (Skelly et al., 2005; Davila et al., 2010; Fox-Powell et al., 2016) have led to recent findings of the presence of water on Mars. This finding suggested that ancient Martian environments could have supported microbial life, and therefore Mars has become the focus of space exploration and life-detection studies (Grotzinger *et al.*, 2014; Grotzinger *et al.*, 2015; Fox-Powell *et al.*, 2016).

To help ensure the legitimacy of life-detection studies and to prevent forward contamination, there are international planetary protection policies restricting the number of microorganisms on spacecraft surfaces, and Special Regions of Mars have been identified where proliferation of known microbes could take place (Schuerger *et al.*, 2013; Rummel *et al.*, 2014; Rettberg *et al.*, 2016). Hence, it is of concern that extremely resistant microorganisms, including *B. subtilis*, have been detected in spacecraft-associated facilities (Venkateswaran *et al.*, 2014; Checinska *et al.*, 2015; Moissl-Eichinger *et al.*, 2016), and that these organisms (and most importantly, their spores), might pose a threat to the forward contamination of surface terrains, or the search for past or present life on Mars (Fajardo-Cavazos *et al.*, 2008; Horneck *et al.*, 2010; Goetz *et al.*, 2016). In spite of its importance, there is a paucity of experimental data on the molecular mechanisms of spore survival of Earth microorganisms in the Martian environment. Consequently, if we are to design adequate planetary protection measures and prevent forward contamination, it is of utmost importance to expand our knowledge on how microorganisms are able to resist Mars' environmental conditions, and thus, potentially survive on this planet.

In the current study a systematic screening was performed to determine if and how *B. subtilis* spores could survive an average day on Mars. A number of spores of *B. subtilis* strains lacking protective elements and/or DNA repair proteins were exposed to 24 h of simulated Martian surface conditions

with or without 8 h of UV radiation, and spore survival and mutagenesis were measured. The results of this study reveal the molecular mechanisms behind *B. subtilis* spore resistance in a Martian environment and assess the possibility of microbial contamination due to spores on the Martian surface.

2 Materials and Methods

2.1 Bacterial strains, growth, sporulation and spore purification

The two sets of *B. subtilis* strains used in this work are listed in Tables 1 and 2, and all are isogenic with their respective wild-type strains, either PS832, PY79 or 168. One set of spores was chosen to determine the role of various spore protection mechanisms, including SASP, Ca-DPA, the spore core hydration level and the spore coat and crust, in spore survival (Table 1); the other set was used to study the importance of different DNA repair mechanisms (Table 2).

The *ligD ku* genes were deleted in strain 168. The deletion cassette was constructed using the oligonucleotide pairs KK294 / 295 (5’-CCGAGCGCCTACGAGGAATTTGTATCGCAACCCGCAAGACGAACCGCTTAG/5‘-CGATGATGGCAGCAAAGACCGCACT), KG297 / KG298 (5’-CCTATCACCTCAAATGGTTCGCTGCTTTAGTGTGAAGAGAAGGAGTACGATTCATG/5’-GCGATATCTCCAAAAGACGGGACGGA) and kan-fwd / kan-rev (5’-CAGCGAACCATTTGAGGTGATAGG/5’-CGATACAAATTCCTCGTAGGCGCTCGG) which were used to amplify the flanking regions and the *aphA3* kanamycin resistance gene. The deletion cassette was used to transform *B. subtilis* using a previously described protocol (Kunst and Rapoport, 1995). Transformants were selected on LB agar plates supplemented with 10 µg mL^{-1} kanamycin. The resulting strain was designated as BP141.

Spores were obtained by cultivation under vigorous aeration at 37 °C for 7 days in double-strength liquid Schaeffer’s sporulation medium (SSM) (Schaeffer *et al.*, 1965) and in a few cases with DPA added to 100 µg mL^{-1}. Spores were purified and stored as described previously (Moeller *et al.*, 2006). Antibiotics (i.e., chloramphenicol (5 µg mL^{-1}), neomycin (10 µg mL^{-1}), spectinomycin (100 µg mL^{-1}), erythromycin (1 µg mL^{-1}), or tetracycline (10 µg mL^{-1})) were used when needed (Paidhungat *et al.*, 2000) (Tables 1 and 2). Final spore suspensions consisted of single spores with no detectable clumps, and were free (> 99 %) of vegetative cells, germinated spores, or cellular debris, as seen in phase-contrast microscopy (data not shown).

2.2 Sample preparation

Spore suspensions were prepared in sterile distilled water such that a 50 µL aliquot contained 5×10^8 spores. Each sample for exposure was prepared by applying 50 µl of spores onto a 10 mm × 20 mm aluminum coupon (Model M4985, Seton, Inc., Branford, CT, USA) to ensure that the spores spread homogenously on the coupons by complete covering of the surface, yielding spore multilayer samples with a thickness of ~ 25 spore layers (Tauscher *et al.*, 2006). In our study, coupons were chosen to simulate surface materials of a spore-contaminated spacecraft. Each set of spore samples was tested in three replicates of each genotype with the same spore concentration. Spore samples were air-dried

under ambient laboratory conditions (20 °C, 33 ± 5 % relative humidity) for 1 d prior to exposure to simulated Mars surface conditions.

2.3 Spore exposure in the Mars simulation chamber

Spore-inoculated coupons were exposed for 24 h to simulated Martian conditions in a cylindrical Mars Simulation Chamber (MSC) (50 cm in diameter by 70 cm long) with a regimen of 8 h simulated Martian solar irradiation exposure and 16 h exposure in the dark. The UVC (200-280 nm) flux on spores in the MSC was measured as 4.04 W m^{-2}, which converts to 14.4 kJ m^{-2} h^{-1} (or 115 kJ m^{-2} d^{-1}) (Table 3). During the 8 h of simulated Martian solar irradiation, one sample set was exposed to full Martian UV conditions [designated as M(+)UV] and the other sample set was covered with aluminum foil, which shielded all applied photonic energy [designated M(-)UV]. The overall simulated Martian conditions of temperature, pressure, and gas composition inside the chamber are listed in **Table 3**. Regarding irradiation conditions, the 8 h of radiation exposure represents a worst-case scenario for high UV flux (note that no ionizing radiation was simulated), and thus likely to give the maximum UV effects on *B. subtilis* spores under Martian conditions. In parallel, two additional sample sets were prepared; one was stored for the same time under ambient laboratory conditions (Earth atmosphere, pressure, room temperature, and protected from light) and the remaining sample set was stored at 4 °C in a refrigerator. The MSC was developed as part of an ongoing series of Mars astrobiology and planetary protection projects, and has been described previously (Schuerger et al., 2008; Schuerger et al., 2011).

2.4 Spore recovery and survival assay

To recover *B. subtilis* spores from aluminum coupons, spore layers were covered by a 10% aqueous polyvinyl alcohol solution (PVA) and after drying the spore-PVA layers were removed as described (Horneck *et al.*, 2001), and suspended in 1 ml of sterile distilled water, resulting in > 95% recovery of the spores (data not shown). The PVA procedure has no geno- or cytotoxic effect on the spore viability (Horneck et al. 2001). Spore survival was determined from serial dilutions in distilled water as colony-forming units after incubation overnight at 37 °C on nutrient broth (NB) agar plates (Difco, Detroit, USA) (Moeller *et al.*, 2007c; Moeller *et al.*, 2010). Spore survival was determined by observing standard colony formation of macroscopic visible colonies on NB agar containing the appropriate selective antibiotic, as described above (Horneck *et al.*, 2001). The relative sensitivity of spores of each mutant strain was determined with respect to that of the corresponding wild-type spores, and in some cases with *splB* spores, results were compared statistically using the Student's *t*-test and differences with P values of ≤ 0.05 were considered statistically significant.

2.5 Detection of sporulation deficiency

To verify mutation induction caused by exposure to Martian conditions, 250 *B. subtilis* colonies arising from survivors of each Martian exposure tested were picked and streak-purified on SSM-agar plates solidified with 1.5% agar, containing the appropriate antibiotic(s), and incubated at 37 °C for 7 d. Sporulation deficiencies were determined visually by changes in colony morphology and pigmentation. Sporulated *B. subtilis* colonies show brownish pigmentation after extended incubation on sporulation plates, whereas a decrease in pigmentation and a translucent appearance are characteristic of asporogenous or Spo *B. subtilis* mutants (Piggot and Coote 1976; Hullo et al., 2001; Fajardo-Cavazos

et al., 2005). The frequency of Spo$^-$ mutants was expressed as the ratio of the Spo$^-$ colonies to the total 250 colonies picked after 7 days of incubation on SMM plates. To verify the Spo$^-$ mutation rates, plate from spores that had been exposed in colonies were individually transferred into 5 mL of SSM media and incubated for 24 h at 37 °C. Sporulation was then induced by diluting the overnight culture 1:100 into 5 mL of SSM medium. To determine the number of spores formed, after 24 h of cultivation, appropriate dilutions of cultures were plated on NB agar before and after a heat-shock (80 °C; 10 min) to kill growing or sporulating cells but not spores, as described (Maughan *et al.*, 2007). Each analysis of the selected Spo$^-$ mutants was repeated at least three times.

2.6 Numerical and statistical analysis

The surviving fraction of *B. subtilis* spores was determined from the quotient N/N_0, with N = the number of colony-forming units (CFU) of the Mars-exposed sample and N_0 that of the untreated controls. The Spo$^-$ mutant frequencies from the control and M(+/-)UV exposed spores were determined from three replicate samples. The frequency of Spo$^-$ mutations in samples induced by exposure to the M(+/-)UV conditions was determined as [M/N] - m_S], with M = the total number of mutants from the exposed samples; N=250; and m_S = frequency of spontaneous Spo$^-$ mutations in unexposed samples. The sporulation frequency of the induced asporogenous mutants was determined by dividing the CFU after heat shock (spores) by the CFU before heat shock (growing/sporulating cells and spores). The data shown are expressed as averages ± standard deviations, and results were compared statistically using the Student's *t*-test. Values were analyzed in multigroup pairwise combinations, and differences with *P* values of ≤ 0.05 were considered statistically significant (Moeller *et al.*, 2005; Moeller *et al.*, 2006; Moeller *et al.*, 2007a; Moeller *et al.*, 2007c; Horneck *et al.*, 2008; Moeller *et al.*, 2008) .

3 Results

To know which spore components and molecular mechanisms are involved in *B. subtilis* spore resistance to simulated Mars surface conditions, two sets of *B. subtilis* spores were exposed to a simulated Martian atmospheric environment with or without 8 h of UV radiation (M(+/-)UV). The first set comprised spores deficient in spore protective components (Table 1), and the second set comprised spores deficient in various DNA repair mechanisms (Table 2). A summary registering which mutant genotypes, and respective missing mechanisms of protection or repair, revealed the highest and/or lowest sensivity to M(+/-)UV tested conditions is presented in Figure 4.

3.1 Spore protection

When exposed to both M(+/-)UV conditions, *B. subtilis* spores lacking proteins responsible for spore coat assembly were significantly more sensitive than wild-type spores (Table 4, Figure S01, S02, S03). The outer and inner spore coats provided significant protection against the Martian environment, with *cotE* PY79 spores, lacking the outer coat, being less sensitive [15-fold M(+)UV and 18-fold M(-)UV] than *safA* spores, lacking the inner spore coat [~ 240-fold M(+)UV and 63-fold M(–)UV], when compared with the wild-type spores. Spores lacking both outer and inner spore coat layers (*cotE safA* spores) exhibited astonishing increases in sensitivity of ~ 1000-fold in M(+)UV, and ~ 200-fold in M(-)UV, compared to wild-type spores (Table 4, Figure S01, S02, S03). Despite the striking effects of inner and outer coat defects on spore resistance to M(+/-)UV, the loss of the spore crust layer (*cotVW,*

and *cotX cotYZ* spores) had no significant effects on spore survival under the tested conditions (Table 4, Figure S01, S02, S03).

A second group of crucial protective components in spores is the α/β-type SASP that saturate spore DNA and protect it from damage. Spores lacking SASP-α and -β (*sspA sspB* spores) are thus lacking ~80 % of the α/β-type SASP pool (Hathout et al., 2003). When exposed to M(+/-)UV *sspA sspB* spores had increased sensitivity when compared with the wild-type, being significantly more sensitive to M (+)UV (273-fold, with a *P* value of 0.0015) than to M(-)UV (17-fold, with a *P* value of 0.0021) (Table 4, Figure S04, S05, S06, S07). Interestingly, *sspE* spores, which lack the most prominent SASP, SspE), had no significant effect on spore survival in both M(+/-)UV (with a *P* value of 0.4936, same as wild-type), but had increased sensitivity when additionally lacking SASP-α and -β (*sspE sspA sspB* spores). Results show *sspE sspA sspB* spores with 435-fold and 39-fold sensitivity in M(+)UV (with a *P* value of 0.0012) and M(-)UV (with a *P* value of 0.0013), respectively, when compared with wild-type spores (Table 4, Figure S04, S05, S06, S07).

A third spore protective factor is the low water content in the spore core. Spores with higher core water content (*dacB,* and *sleB spoVF* spores) exhibited lower resistance to conditions M(+/-)UV, when compared to wild-type spores (Table 4, Figure S04, S05, S06, S07). Notably, spores lacking α/β-type SASP and either DacB (*dacB sspA sspB* spores, with a *P* value of 0.0086) or CaDPA (*sleB spoV sspA sspB* spores, with a *P* value of 0.0053) were more sensitive to the Martian environment than either *dacB* or *sleB spoVF* spores. Results also show that addition of DPA to the sporulation medium suppressed *sleB spoVF* spores' decreased resistance while sporulating, reaching near wild-type survivability levels (Table 4, Figure S04, S05, S06, S07).

Spores lacking an outer coat with an additional SASP deficiency (*cotE sspA sspB* spores, with a *P* value of 0.0052), were more sensitive to Mars conditions than spores lacking either protective component alone (*cotE*, and *sspA sspB* spores) (Table 4, Figure S04, S05, S06, S07). An additional deficiency in Ca-DPA (*cotE sleB spoVF sspA sspB* spores), and consequent higher core water content, resulted in rapid killing with a 10^5-fold, in M(+)UV and 10^4-fold in M(-)UV, greater sensitivity compared with the wild-type (with *P* values of 0.0001 or 0.0001, respectively). However, the effects of the *sleB spoVF* mutations were again suppressed when these spores were prepared with DPA added to the sporulation medium with 10^3-fold greater sensitivity compared with the wild-type in M(+)UV and 646-fold in M(-)UV (Table 4, Figure S04, S05, S06, S07).

3.2 Spore DNA repair

Bacillus subtilis spores rely on a complex network of mechanisms to repair DNA damage accumulated during periods of dormancy, and ensure genomic integrity. When spores were exposed to M(+)UV, SP lyase deficient spores (*splB* spores, with a *P* values of 0.0004) were ~300-fold more sensitive than wild-type spores, whereas spores lacking NHEJ (*ligD ku*, with a *P* values of 0.0049) or HR (*recA*, with a *P* values of 0.0037) were only ~ 35 and ~ 80-fold more sensitive than wild-type spores (Table 5, Figure S08, S09, S10, S11, S12). A number of single or double mutations in other DNA repair genes resulted in smaller amounts of sensitization of spores to M(+/-)UV, including *exoA nfo*, *uvrAB*, *mfd*, *sbcDC*, *polY1 polY2,* and *mutSL* mutations. Mutation of the *disA* gene (lacking DNA integrity scanning protein) had only minimal (but not significant) effects on spore survival in M(+/-)UV reaching near

wild-type levels of survivability (with a *P* value of 0.0943). Sensitivity of *recA* and *ligD ku* mutant spores was revealed to be in the same order of magnitude in both tested environments M(+/-)UV, being of ~ 80-90-fold for *recA* spores in M(+)UV, and ~ 30-fold for *ligD ku* in M(+)UV and M(-)UV (Table 5, Figure S08, S09, S10, S11, S12).

Analysis of the M(+/-)UV survival rates of *splB* spores additionally lacking other DNA repair genes, revealed that almost all DNA repair mutations caused significant increases in spore sensitivity, when compared to that of the *splB* single mutant spores. Spores lacking SP lyase (SP repair) and strand specific DNA repair (*mfd splB* spores, with a *P* value of 0.0001) as well as spores lacking SP repair and NER (*uvrAB splB* spores, with a *P* value of 0.0001) exhibited dramatic increases in M(+)UV sensitivity of 60- and 250-fold, respectively (Table 5, Figure S13).

3.3 Sporulation deficiency

When assessing sporulation deficiency through mutagenesis in survivors of spores of various strains after M(+/-)UV exposure, ~ 3% of survivors of wild-type spores exposed to M(-)UV had accumulated Spo^- mutants. Under Mars(+)UV conditions, spore components exhibited importance in mutagenesis, in order from highest to lowest frequency: major SASPs > intact spore coats > DPA > reduced spore core water. Spo^- ratio (calculated as in Materials and Methods section 2.5 and 2.6) was similar to that for *disA*, *ywjD* and *splB* spores exposed to M(-)UV. Survivors of all other DNA repair mutant strains exposed to M(-)UV exhibited increased mutagenesis, from ~ 20 % in *ligD ku* spores to ~ 30 % in *exoA nfo* and *mutSL* spores. As expected, levels of Spo^- mutants were increased with M(+)UV exposure of spores of all DNA repair mutants, reaching ~ 60 % in *mutSL* spores that lack the ability to repair DNA via mismatch repair (Figure 2, 3). Additional inactivation of *splB* with other repair mechanisms did not result in higher mutagenicity neither under UV exposed or UV-shielded conditions.

4 Discussion

Because of our extensive understanding of the genetics and molecular biology of *B. subtilis* spore protection and repair mechanisms, these spores are of great value in investigating spore resistance to extreme environments, methods for sterilization and disinfection, and in verifying planetary protection protocols. *B. subtilis* spore survival in a simulated Martian surface environment is dependent on complex systems that rely on two different strategies: "damage prevention" and "damage repair".

The current study demonstrates that, when exposed to M(+)UV, *B. subtilis* spore survival was dependent on the ability to maintain spore core dehydration; to effectively protect spore DNA through binding of major α/β-type SASP, and that spore damage by Martian UV generates primarily SP. However, when exposed to M(-)UV, the most important factor in spore survival was the multilayered spore coat, as seen with *cotE safA* spores (Figure 4); and lethal damage by M(-)UV was largely due to DNA as seen by increased M(-)UV sensitivity of *recA* spores (Figure 4).

4.1 Spore outer coat as the most important protection mechanism

Altogether, the ability of *B. subtilis* spores to maximally survive M(+/-)UV was due to the ability to prevent DNA damage through several protective components, including the coat, low core water content, Ca-DPA and α/β-type SASP. Yet, it was the spore outer coat (*cotE*) that was the most important protection mechanism, as spores lacking DPA, α/β-type SASP, and outer coat (*cotE sleB*

spoVF sspA sspB spores) were 100-fold more sensitive than spores lacking DPA and α/β-type SASP, but with an intact outer coat (*sspA sspB sleB spoVF* spores). The latter observation is consistent with previous work showing that the spore coat is important for spore resistance to solar radiation, particularly UV-B and UV-A (Riesenman and Nicholson, 2000; Moeller *et al.*, 2014). It is notable that the *B. subtilis* spore crust, the spore's outermost layer played no significant role in spore resistance to M(+/-)UV. The precise role of the spore crust in spore properties and the detailed role of individual spore crust proteins is not yet well understood. This is particularly important as individual crust mutants were recently suggested to yield different phenotypes with respect to the double crust mutant spores (tested in this study) (McKenney and Eichenberger, 2012; Krajcikova *et al.*, 2017).

4.2 Spore decreased core water content is key for survival

During spore formation, spore core dehydration and mineralization is established, in part, as DPA is taken up into the forespore and chelates Ca^{2+} ions (Ca-DPA), displacing core water (reviewed in Setlow, 2014). The compression of the forespore that takes place later in sporulation also displaces significant amounts of core water, further reducing core water content (Magge *et al.*, 2008). How the latter takes place is not known, but likely involves the spore cortex peptidoglycan in some fashion (Zhang et al., 2012). In the current study, two different core water deficiencies were tested: (1) *dacB* spores, which have an altered cortex and thus present elevated core water levels and (2) *sleB spoVF* spores, which lack Ca-DPA due to the *spoVF* mutation, are stabilized against spontaneous spore germination by the *sleB* mutation, and have elevated core water because Ca-DPA has been replaced by water (Paidhungat *et al.*, 2001). Results demonstrated *dacB* spores to be significantly more sensitive in both M(+/-)UV, when compared with the wild-type spores. This increased sensitivity was perhaps due to greater molecular damage (at least some to DNA) induced by oxidative stress when in the high vacuum/desiccation of the Martian environment. Calcium-DPA-deficient *sleB spoVF* spores were shown to be more sensitive to M(+/-)UV than Ca-DPA-replete spores, in particular to exposed to M(+)UV, as shown previously (Setlow *et al.*, 2006; Magge *et al.*, 2008). This happens because *sleB spoVF* sporulating cells are unable to synthesize DPA, but exogenously added DPA can enter the spore, reaching near wild-type levels. Although, whether the latter effects are due only to the spore elevated core water content, or to some direct protective effect of Ca-DPA is not clear.

4.3 SspE may provide some protection when SspA and SspB are missing

Protection of the DNA in the spore core is also dependent on the high levels of α/β-type small, acid-soluble spore proteins (SASP) (Magge *et al.*, 2008). These act by saturating spore DNA, and are extremely important when exposed to desiccation and UV radiation (Mason and Setlow, 1986; Moeller *et al.*, 2008). As expected, spores lacking SASP-α and -β (*sspA sspB* spores), and thus lacking ~80 % of the α/β-type SASP pool (Hathout et al., 2003), had increased sensitivity to both M(+/-)UV (when compared with the wild-type), being significantly more sensitive to UV-irradiated, rather than to non-irradiated Martian environments. In contrast, *sspE* mutants lacking the most prominent SASP, SspE, which bounds poorly to DNA in wild-type spores, had no significant effect on spore survival in both M(+/-)UV. However, SspE may provide some protection when SspA and SspB are missing, as suggested by the increased sensitivity in *sspE sspA sspB* spores, when compared with *sspA sspB* mutants. Removing α/β-type SASP in spore coat- or cortex-defective spores (*cotE sspA sspB; dacB sspA sspB and sleB spoVF sspA sspB* spores) increased spore sensitivity to M(+/-)UV, confirming

DNA-binding α/β-type SASP as a key factor in *B. subtilis* spore resistance to M(+/-)UV, presumably by the α/β-type SASP binding to spore DNA and converting the spore chromosome into a monogenomic toroidal shaped A-DNA structure (Setlow and Li, 2015).

4.4 Double Strand Breaks and base modifications in M(-)UV

The UV-exposed Martian surface conditions have direct and indirect effects on cells, either through the direct transfer of radiation energy, and consequent damage of biomolecules or through generation of reactive nitrogen species (RNS), or reactive oxygen species (ROS) that then induce biomolecular damage (Lenhart *et al.*, 2012). Ultraviolet-induced damage is typically seen as DNA SSB or DSB, as well as photolesions such as CPDs, 6-4 PPs or SP (Setlow and Li, 2015). Spores lacking HR (*recA*), NHEJ (*lig ku*), or BER (*exoA nfo*) were significantly more sensitive to M(-)UV than wild-type spores (Figure 5), indicating that DSB and base modifications comprise a substantial fraction of the DNA damage suffered, likely due to the extreme desiccation in M(-)UV (Rebeil *et al.*, 1998; Setlow and Li, 2015; Nicholson *et al.*, 2018).

4.5 Spore Photoproduct as major damage in M(+)UV

The formation of SP as a major product of UV-damage with M(+)UV exposure was expected, and has been shown previously (Xue and Nicholson, 1996). Accumulated SPs have been shown to be repaired by SP lyase (SPL), and also by the NER pathway - mechanisms that are crucial in spore UV resistance (Setlow and Li, 2015). The current study is the first to analyze the relative sensitivities of various SP repair mutant strains of *B. subtilis* spores to the Martian environment, including results with spores lacking other DNA repair mechanisms. Notably, in M(-)UV accumulated SP in spores exposed to M(+)UV were shown to be repaired by both SplB and the NER pathway, mechanisms that are crucial in spore resistance to natural UV environments (Xue and Nicholson, 1996; Setlow and Li 2015).

4.6 YwdJ and Mfd might participate in SP repair

In the current study, *ywjD* spores lacking the UV-damage endonuclease YwjD, showed no increased sensitivity to M(+/-)UV. Yet, *ywjD splB* spores were more sensitive to M(+)UV than *splB* single mutant spores. This suggests that YwjD might participate in SP repair, functioning as an alternative DNA repair enzyme, and is in line with previous studies (Ramirez-Guadiana et al., 2012). While *ywjD* spores showed no increased sensitivity to M(+/-)UV, spores were more sensitive to M(+)UV than *splB* spores, suggesting that YwdJ can also participate in SP repair. Moreover, *mfd* s*plB* spores, lacking both SP lyase and the spores also much to M(+)UV *splB* spores indicating that transcription, had also increased sensitivity to M(+)UV, when compared with *splB* single mutant spores. Thus, transcription-coupled repair might be involved in SP repair. This is likely due to the role Mfd plays in NER (Gomez-Marroquin *et al.*, 2016). The lack of the DNA exonuclease SbcDC involved in inter-strand cross-link repair (ISCLR) (Mascarenhas *et al.*, 2006; Lenhart *et al.*, 2012) also demonstrated increased sensitivity in M(+)UV (*sbcDC splB* spores), when compared with *splB* single mutant spores. This was not observed, however, in *polY1 polY2* spores, lacking both DNA polymerases PolY1 and PolY2, which mediate DNA repair by translesion synthesis.

4.7 Sporulation deficiency

Strains lacking *mutSL* or *exoA nfo* shown an increased Spo$^-$ rate after exposure to M(+/-)UV, suggesting their critical involvement of MMR and BER in DNA repair in order to ensure sporulation. An increased loss of viability during sporulation of strains lacking the ability to repair DNA damage by mismatch repair had already been suggested (Modrich, 1996; Salas-Pacheco *et al.*, 2005; Ibarra *et al.*, 2008; Fukui, 2010), indicating *mutSL* contribution to genome stability and overall spore resistance. In turn, *exoA nfo* genes are known to encode for apurinic/apyrimidinic endonucleases involved in the repair of oxidative DNA damage through BER (Ibarra *et al.*, 2008; Moeller *et al.*, 2011; Campos *et al.*, 2014). This means that spores exposed to M(+/-)UV, ensure sporulation through efficient mismatch repair by mutSL, and repair oxidative damage by BER (*exoA nfo*). Especially, the absence of the proteins LigD, Ku, ExoA, Nfo, SbcDC, and MutSL showed significant increased mutation frequencies of Spo$^-$, indicating their crucial role in DNA repair, genome stability and restoration. In the current study however, the interaction between Nfo and ExoA and the DNA integrity scanning protein DisA (Campos *et al.*, 2014) was not assessed, and would be advised for future studies on the process of oxidative DNA damage repair after exposure to simulated Martian conditions (Campos *et al.*, 2014). This sporulation deficiency analysis is informative on the types of error-free or error-prone mechanisms leading to spore survival. For instance, Figure 2 shows that RecA-mediated homologous recombination (HR) and wild type have similar proportions of Spo- mutants, indicating that spore survival in a *recA*-mutant is error-free. Considering that other repair mechanisms such as SP, NER, NHEJ and MMR are still at least partially functional in a *recA*-deficient background, this is the best argument presented in the paper to say that UV-induced photolesions such as DNA strand breaks, dimers or AP sites are the major lesion caused by Martian exposure.

4.8 Conclusion

When considering a Mars exploration scenario one can expect spore killing by the Martian environment to be mostly UV-driven, as the other environmental conditions (atmospheric composition, low pressure and low temperature) were shown to have only minimal effects on wild-type spore viability. Most importantly, the current study demonstrates that wild-type *B. subtilis* spores could survive in a Mars surface environment, if somehow shielded from UV (e.g., by dust, rocks, or spacecraft surface irregularities) It should be noted, however, that this study determined survivability by the ability to form colony forming units, and any defects in growth after exposure were not analyzed. Besides, increased spore sensitivity has been reported when in contact with Mars analogue soils (Schuerger *et al.*, 2003; Moeller *et al.*, 2010); and vegetative cells of *B. subtilis* were found to be more sensitive the presence of perchlorates (found in Mars subglacial brines) irradiated with a Martian UV-flux (Wadsworth and Cockell, 2017). Thus, future efforts should focus on assessing spore survival and viability in real long-duration Mars mission scenarios. This can be done by: (1) directly determining DNA damage in wild-type spores exposed to M(+/-)UV, (2) address whether exposed mutants have growth defects, after germination, (3) taking into consideration the shielding of spores via Mars regolith and other relevant materials, and (4) assess the effect of Mars surface photochemistry on spore sensitivity.

5 Conflict of Interest

The authors declare that the research was conducted in the absence of any commercial or financial relationships that could be construed as a potential conflict of interest.

6 Author Contributions

The study was conceived by RM. Experiments were conducted by MC, FMF, FMC, RM and PE. The simulation experiments in the described Mars chamber were conducted by ACS. The manuscript was written by MC with input from RM, FMF, FMC, WLN, ACS, PE and PS.

7 Funding

MC, FMF and RM were supported by the DLR grant FuE-Projekt ISS LIFE, Programm RF-FuW, Teilprogramm 475. MC was supported by the DLR/DAAD Research Fellowship – Doctoral Studies in Germany, 2017 (57370122). FMC was supported by the DFG grant Co 1139/1-2. RM was supported by a DLR grant "*Forschungssemester*". FMF was supported by the Helmholtz Space Life Sciences Research School SpaceLife PhD fellowship. ACS and WLN were supported by NASA Planetary Protection Office grants (ASC: NNX12AJ84G, NNA05CS68G, and WLN: NNA05CS68G).

8 Acknowledgments

We acknowledge Andrea Schröder, Marina Raguse and Katja Nagler for their assistance. We thank Mario Pedraza-Reyes and Jörg Stülke for cordially providing several *B. subtilis* strains.

9 References

Campos, S.S., Ibarra-Rodriguez, J.R., Barajas-Ornelas, R.C., Ramirez-Guadiana, F.H., Obregon-Herrera, A., Setlow, P., *et al.* (2014). Interaction of apurinic/apyrimidinic endonucleases Nfo and ExoA with the DNA integrity scanning protein DisA in the processing of oxidative DNA damage during *Bacillus subtilis* spore outgrowth. *J. Bacteriol.* 196**,** 568-578.

Checinska, A., Probst, A.J., Vaishampayan, P., White, J.R., Kumar, D., Stepanov, V.G., *et al.* (2015). Microbiomes of the dust particles collected from the International Space Station and Spacecraft Assembly Facilities. *Microbiome* 3**,** 50.

Davila, A.F., Skidmore, M., Fairen, A.G., Cockell, C., and Schulze-Makuch, D. (2010). New priorities in the robotic exploration of Mars: the case for in situ search for extant life. *Astrobiology* 10**,** 705-710.

Djouiai, B., Thwaite, J.E., Laws, T.R., Commichau, F.M., Setlow, B., Setlow, P., *et al.* (2018). Role of DNA repair and protective components in *Bacillus subtilis* spore resistance to inactivation by 400 nm blue light. *Appl. Environ. Microbiol.* (pii: AEM.01604-18. doi: 10.1128/AEM.01604-18).

Dose, K., Bieger-Dose, A., Dillmann, R., Gill, M., Kerz, O., Klein, A., *et al.* (1995). ERA-experiment "Space Biochemistry". *Adv. Space. Res.* 16**,** 119-129.

Duigou, S., Ehrlich, S.D., Noirot, P., and Noirot-Gros, M.F. (2005). DNA polymerase I acts in translesion synthesis mediated by the Y-polymerases in *Bacillus subtilis*. *Mol. Microbiol.* 57**,** 678-690.

Eichenberger, P., Fujita, M., Jensen, S.T., Conlon, E.M., Rudner, D.Z., Wang, S.T.*, et al.* (2004). The program of gene transcription for a single differentiating cell type during sporulation in *Bacillus subtilis*. *PLoS Biol.* 2**,** e328.

Fajardo-Cavazos, P., Schuerger, A.C., and Nicholson, W.L. (2008). Persistence of biomarker ATP and ATP-generating capability in bacterial cells and spores contaminating spacecraft materials under earth conditions and in a simulated martian environment. *Appl. Environ. Microbiol.* 74**,** 5159-5167.

Fajardo-Cavazos, P., Schuerger, A.C., and Nicholson, W.L. (2010). Exposure of DNA and *Bacillus subtilis* spores to simulated martian environments: use of quantitative PCR (qPCR) to measure inactivation rates of DNA to function as a template molecule. *Astrobiology* 10**,** 403-411.

Fox-Powell, M.G., Hallsworth, J.E., Cousins, C.R., and Cockell, C.S. (2016). Ionic strength is a barrier to the habitability of Mars. *Astrobiology* 16**,** 427-442.

Fukui, K. (2010). DNA mismatch repair in eukaryotes and bacteria. *J. Nucleic Acids* 2010.

Gargaud, M., Amils, R., and Cleaves, H.J. (2011). *Encyclopedia of Astrobiology.* Springer.

Goetz, W., Brinckerhoff, W.B., Arevalo, R., Freissinet, C., Getty, S., Glavin, D.P.*, et al.* (2016). MOMA: the challenge to search for organics and biosignatures on Mars. *Int. J. Astrobiol.* 15**,** 239-250.

Gomez-Marroquin, M., Martin, H.A., Pepper, A., Girard, M.E., Kidman, A.A., Vallin, C.*, et al.* (2016). Stationary-phase mutagenesis in stressed *Bacillus subtilis* cells operates by mfd-dependent mutagenic pathways. *Genes (Basel)* 7**,** 1-13.

Grotzinger, J.P., Gupta, S., Malin, M.C., Rubin, D.M., Schieber, J., Siebach, K.*, et al.* (2015). Deposition, exhumation, and paleoclimate of an ancient lake deposit, Gale crater, Mars. *Science* 350**,** aac7575 7571-7512.

Grotzinger, J.P., Sumner, D.Y., Kah, L.C., Stack, K., Gupta, S., Edgar, L.*, et al.* (2014). A habitable fluvio-lacustrine environment at Yellowknife Bay, Gale crater, Mars. *Science* 343**,** 1242777.

Gunka, K., Tholen, S., Gerwig, J., Herzberg, C., Stulke, J., and Commichau, F.M. (2012). A high-frequency mutation in *Bacillus subtilis*: requirements for the decryptification of the *gudB* glutamate dehydrogenase gene. *J. Bacteriol.* 194**,** 1036-1044.

Guo, J., Zeitlin, C., Wimmer-Schweingruber, R.F., Mcdole, T., Kühl, P., Appel, J.C.*, et al.* (2018). A generalized approach to model the spectra and radiation dose rate of Solar Particle Events on the surface of Mars. *Astron. J.* 155**,** 49.

Hackett, B.H., and Setlow, P. (1988). Properties of spores of *Bacillus subtilis* strains which lack the major small, acid-soluble protein. *J. Bacteriol.* 170**,** 1403-1404.

Horneck, G., Klaus, D.M., and Mancinelli, R.L. (2010). Space microbiology. *Microbiol. Mol. Biol. Rev.* 74**,** 121-156.

Horneck, G., Rettberg, P., Reitz, G., Wehner, J., Eschweiler, U., Strauch, K.*, et al.* (2001). Protection of bacterial spores in space, a contribution to the discussion on Panspermia. *Orig. Life. Evol. Biosph.* 31**,** 527-547.

Horneck, G., Stoffler, D., Ott, S., Hornemann, U., Cockell, C.S., Moeller, R.*, et al.* (2008). Microbial rock inhabitants survive hypervelocity impacts on Mars-like host planets: first phase of lithopanspermia experimentally tested. *Astrobiology* 8**,** 17-44.

Ibarra, J.R., Orozco, A.D., Rojas, J.A., Lopez, K., Setlow, P., Yasbin, R.E.*, et al.* (2008). Role of the Nfo and ExoA apurinic/apyrimidinic endonucleases in repair of DNA damage during outgrowth of *Bacillus subtilis* spores. *J. Bacteriol.* 190**,** 2031-2038.

Jakosky, B.M., Nealson, K.H., Bakermans, C., Ley, R.E., and Mellon, M.T. (2003). Subfreezing activity of microorganisms and the potential habitability of Mars' polar regions. *Astrobiology* 3, 343-350.

Khodadad, C.L., Wong, G.M., James, L.M., Thakrar, P.J., Lane, M.A., Catechis, J.A.*, et al.* (2017). Stratosphere conditions inactivate bacterial endospores from a Mars spacecraft assembly facility. *Astrobiology* 17**,** 337-350.

Krajcikova, D., Forgac, V., Szabo, A., and Barak, I. (2017). Exploring the interaction network of the *Bacillus subtilis* outer coat and crust proteins. *Microbiol. Res.* 204**,** 72-80.

Kunst, F., and Rapoport, G. (1995). Salt stress is an environmental signal affecting degradative enzyme synthesis in *Bacillus subtilis*. *J. Bacteriol.* 177**,** 2403–2407.

Lenhart, J.S., Schroeder, J.W., Walsh, B.W., and Simmons, L.A. (2012). DNA repair and genome maintenance in *Bacillus subtilis*. *Microbiol. Mol. Biol. Rev.* 76**,** 530-564.

Loshon, C.A., Genest, P.C., Setlow, B., and Setlow, P. (1999). Formaldehyde kills spores of *Bacillus subtilis* by DNA damage and small, acid-soluble spore proteins of the α/β-type protect spores against this DNA damage. *J. Appl. Microbiol.* 87**,** 8–14.

Magge, A., Granger, A.C., Wahome, P.G., Setlow, B., Vepachedu, V.R., Loshon, C.A.*, et al.* (2008). Role of dipicolinic acid in the germination, stability, and viability of spores of *Bacillus subtilis*. *J. Bacteriol.* 190**,** 4798-4807.

Mascarenhas, J., Sanchez, H., Tadesse, S., Kidane, D., Krisnamurthy, M., Alonso, J.C.*, et al.* (2006). *Bacillus subtilis* SbcC protein plays an important role in DNA inter-strand cross-link repair. *BMC Mol. Biol.* 7**,** 20.

Mason, J.M., and Setlow, P. (1986). Essential role of small, acid-soluble spore proteins in resistance of *Bacillus subtilis* spores to UV light. *J. Bacteriol.* 167**,** 174-178.

Maughan, H., Masel, J., Birky, C.W., Jr., and Nicholson, W.L. (2007). The roles of mutation accumulation and selection in loss of sporulation in experimental populations of *Bacillus subtilis*. *Genetics* 177**,** 937-948.

McKenney, P.T., and Eichenberger, P. (2012). Dynamics of spore coat morphogenesis in *Bacillus subtilis*. *Mol. Microbiol.* 83**,** 245-260.

Mehne, F.M., Gunka, K., Eilers, H., Herzberg, C., Kaever, V., and Stulke, J. (2013). Cyclic di-AMP homeostasis in *Bacillus subtilis*: both lack and high level accumulation of the nucleotide are detrimental for cell growth. *J. Biol. Chem.* 288**,** 2004-2017.

Modrich, P. (1996). Mismatch repair in replication fidelity, genetic recombination, and cancer biology. *Annu. Rev. Biochem.* 65**,** 101-133.

Moeller, R., Douki, T., Cadet, J., Stackebrandt, E., Nicholson, W.L., Rettberg, P.*, et al.* (2007a). UV-radiation-induced formation of DNA bipyrimidine photoproducts in *Bacillus subtilis* endospores and their repair during germination. *Int. Microbiol.* 10**,** 39-46.

Moeller, R., Horneck, G., Facius, R., and Stackebrandt, E. (2005). Role of pigmentation in protecting *Bacillus* sp. endospores against environmental UV radiation. *FEMS Microbiol. Ecol.* 51**,** 231-236.

Moeller, R., Horneck, G., Rettberg, P., Mollenkopf, H.J., Stackebrandt, E., and Nicholson, W.L. (2006). A method for extracting RNA from dormant and germinating *Bacillus subtilis* strain 168 endospores. *Curr. Microbiol.* 53**,** 227-231.

Moeller, R., Raguse, M., Reitz, G., Okayasu, R., Li, Z., Klein, S.*, et al.* (2014). Resistance of *Bacillus subtilis* spore DNA to lethal ionizing radiation damage relies primarily on spore core

components and DNA repair, with minor effects of oxygen radical detoxification. *Appl. Environ. Microbiol.* 80**,** 104-109.

Moeller, R., Reitz, G., Li, Z., Klein, S., and Nicholson, W.L. (2012a). Multifactorial resistance of *Bacillus subtilis* spores to high-energy proton radiation: role of spore structural components and the homologous recombination and non-homologous end joining DNA repair pathways. *Astrobiology* 12**,** 1069-1077.

Moeller, R., Rohde, M., and Reitz, G. (2010). Effects of ionizing radiation on the survival of bacterial spores in artificial martian regolith. *Icarus* 206**,** 783-786.

Moeller, R., Schuerger, A.C., Reitz, G., and Nicholson, W.L. (2012b). Protective role of spore structural components in determining *Bacillus subtilis* spore resistance to simulated mars surface conditions. *Appl. Environ. Microbiol.* 78**,** 8849-8853.

Moeller, R., Setlow, P., Horneck, G., Berger, T., Reitz, G., Rettberg, P.*, et al.* (2008). Roles of the major, small, acid-soluble spore proteins and spore-specific and universal DNA repair mechanisms in resistance of *Bacillus subtilis* spores to ionizing radiation from X rays and high-energy charged-particle bombardment. *J. Bacteriol.* 190**,** 1134-1140.

Moeller, R., Setlow, P., Pedraza-Reyes, M., Okayasu, R., Reitz, G., and Nicholson, W.L. (2011). Role of the Nfo and ExoA apurinic/apyrimidinic endonucleases in radiation resistance and radiation-induced mutagenesis of *Bacillus subtilis* spores. *J. Bacteriol.* 193**,** 2875-2879.

Moeller, R., Stackebrandt, E., Reitz, G., Berger, T., Rettberg, P., Doherty, A.J.*, et al.* (2007c). Role of DNA repair by nonhomologous-end joining in *Bacillus subtilis* spore resistance to extreme dryness, mono- and polychromatic UV, and ionizing radiation. *J. Bacteriol.* 189**,** 3306-3311.

Moissl-Eichinger, C., Cockell, C., and Rettberg, P. (2016). Venturing into new realms? Microorganisms in space. *FEMS Microbiol. Rev.* 40**,** 722-737.

Nicholson, W.L., and Schuerger, A.C. (2005). *Bacillus subtilis* spore survival and expression of germination-induced bioluminescence after prolonged incubation under simulated Mars atmospheric pressure and composition: implications for planetary protection and lithopanspermia. *Astrobiology* 5**,** 536-544.

Nicholson, W.L., Schuerger, A.C., and Douki, T. (2018). The photochemistry of unprotected DNA and DNA inside *Bacillus subtilis* spores exposed to simulated Martian surface conditions of atmospheric composition, temperature, pressure, and solar radiation. *Astrobiology* 18**,** 393-402.

Paidhungat, M., Ragkousi, K., and Setlow, P. (2001). Genetic requirements for induction of germination of spores of *Bacillus subtilis* by $Ca(^{2+})$-dipicolinate. *J. Bacteriol.* 183**,** 4886-4893.

Paidhungat, M., Setlow, B., Driks, A., and Setlow, P. (2000). Characterization of spores of *Bacillus subtilis* which lack dipicolinic acid. *J. Bacteriol.* 182**,** 5505–5512.

Popham, D.L., Sengupta, S., and Setlow, P. (1995). Heat, hydrogen peroxide, and UV resistance of *Bacillus subtilis* spores with increased core water content and with or without major DNA-binding proteins. *Appl. Environ. Microb.* 61**,** 3633–3638.

Raguse, M., Fiebrandt, M., B., D., Stapelmann, K., Eichenberger, P., Driks, A.*, et al.* (2016). Understanding of the importance of the spore coat structure and pigmentation in the *Bacillus subtilis* spore resistance to low-pressure plasma sterilization. *J. Phys. D. Appl. Phys.* 49**,** 285401

Ramirez-Guadiana, F.H., Barraza-Salas, M., Ramirez-Ramirez, N., Ortiz-Cortes, M., Setlow, P., and Pedraza-Reyes, M. (2012). Alternative excision repair of ultraviolet B- and C-induced DNA damage in dormant and developing spores of *Bacillus subtilis*. *J. Bacteriol.* 194**,** 6096-6104.

Rebeil, R., Sun, Y., Chooback, L., Pedraza-Reyes, M., Kinsland, C., Begley, T.P.*, et al.* (1998). Spore photoproduct lyase from *Bacillus subtilis* spores is a novel iron-sulfur DNA repair enzyme

which shares features with proteins such as class III anaerobic ribonucleotide reductases and pyruvate-formate lyases. *J. Bacteriol.* 180, 4879-4885.

Rettberg, P., Anesio, A.M., Baker, V.R., Baross, J.A., Cady, S.L., Detsis, E., *et al.* (2016). Planetary protection and Mars special regions - a suggestion for updating the definition. *Astrobiology* 16, 119-125.

Riesenman, P.J., and Nicholson, W.L. (2000). Role of the spore coat layers in *Bacillus subtilis s*pore resistance to hydrogen peroxide, artificial UV-C, UV-B, and solar UV radiation. *Appl. Environ. Microbiol.* 66, 620–626.

Rivas-Castillo, A.M., Yasbin, R.E., Robleto, E., Nicholson, W.L., and Pedraza-Reyes, M. (2010). Role of the Y-family DNA polymerases YqjH and YqjW in protecting sporulating *Bacillus subtilis* cells from DNA damage. *Curr. Microbiol.* 60, 263-267.

Rummel, J.D., Beaty, D.W., Jones, M.A., Bakermans, C., Barlow, N.G., Boston, P.J., *et al.* (2014). A new analysis of Mars "Special Regions": findings of the second MEPAG Special Regions Science Analysis Group (SR-SAG2). *Astrobiology* 14, 887-968.

Salas-Pacheco, J.M., Setlow, B., Setlow, P., and Pedraza-Reyes, M. (2005). Role of the Nfo (YqfS) and ExoA apurinic/apyrimidinic endonucleases in protecting *Bacillus subtilis* spores from DNA damage. *J. Bacteriol.* 187, 7374-7381.

Schaeffer, P., Millet, J., and Aubert, J.P. (1965). Catabolic repression of bacterial sporulation. *Proc Natl Acad Sci U S A.* 54, 704-711.

Schuerger, A., Richards, J.T., Hintze, P.E., and Kern, R.G. (2005). Surface characteristics of spacecraft components affect the aggregation of microorganisms and may lead to different survival rates of bacteria on Mars landers. *Astrobiology* 5, 545-559.

Schuerger, A.C., Clausen, C., and Britt, D. (2011). Methane evolution from UV-irradiated spacecraft materials under simulated martian conditions: Implications for the Mars Science Laboratory (MSL) mission. *Icarus* 213, 393-403.

Schuerger, A.C., Fajardo-Cavazos, P., Clausen, C.A., Moores, J.E., Smith, P.H., and Nicholson, W.L. (2008). Slow degradation of ATP in simulated martian environments suggests long residence times for the biosignature molecule on spacecraft surfaces on Mars. *Icarus* 194, 86-100.

Schuerger, A.C., Mancinelli, R.L., Kern, R.G., L.J., R., and Mckay, C.P. (2003). Survival of endospores of *Bacillus subtilis* on spacecraft surfaces under simulated martian environments: implications for the forward contamination of Mars. *Icarus* 165, 253-276.

Schuerger, A.C., Ming, D.W., and Golden, D.C. (2017). Biotoxicity of Mars soils: 2. Survival of *Bacillus subtilis* and *Enterococcus faecalis* in aqueous extracts derived from six Mars analog soils. *Icarus* 290, 215-223.

Schuerger, A.C., Ulrich, R., Berry, B.J., and Nicholson, W.L. (2013). Growth of *Serratia liquefaciens* under 7 mbar, 0 degrees C, and CO_2-enriched anoxic atmospheres. *Astrobiology* 13, 115-131.

Setlow, B., Atluri, S., Kitchel, R., Koziol-Dube, K., and Setlow, P. (2006). Role of dipicolinic acid in resistance and stability of spores of *Bacillus subtilis* with or without DNA-protective alpha/beta-type small acid-soluble proteins. *J. Bacteriol.* 188, 3740-3747.

Setlow, P. (2014). Spore resistance properties. *Microbiol. Spectr.* 2.

Setlow, P., and Li, L. (2015). Photochemistry and photobiology of the spore photoproduct: A 50-year journey. *Photochem. Photobiol.* 91, 1263-1290.

Stapelmann, K., Fiebrandt, M., Raguse, M., Awakowicz, P., Reitz, G., and Moeller, R. (2013). Utilization of low-pressure plasma to inactivate bacterial spores on stainless steel screws. *Astrobiology* 13, 597-606.

Tauscher, C., Schuerger, A.C., and Nicholson, W.L. (2006). Survival and germinability of *Bacillus subtilis* spores exposed to simulated Mars solar radiation: Implications for life detection and planetary protection. *Astrobiology* 6**,** 592-605.

Tovar-Rojo, F., and Setlow, P. (1991). Effects of mutant small, acid-soluble spore proteins from *Bacillus subtilis* on DNA in vivo and in vitro. *J. Bacteriol.* 173**,** 4827-4835.

Venkateswaran, K., Vaishampayan, P., Cisneros, J., Pierson, D.L., Rogers, S.O., and Perry, J. (2014). International Space Station environmental microbiome - microbial inventories of ISS filter debris. *Appl. Microbiol. Biotechnol.* 98**,** 6453-6466.

Wadsworth, J., and Cockell, C.S. (2017). Perchlorates on Mars enhance the bacteriocidal effects of UV light. *Sci. Rep.* 7**,** 4662.

Xue, Y., and Nicholson, W.L. (1996). The two major spore DNA repair pathways, nucleotide excision repair and spore photoproduct lyase, are sufficient for the resistance of *Bacillus subtilis* spores to artificial UV-C and UV-B but not to solar radiation. *Appl. Environ. Microbiol.* 62**,** 2221-2227.

Young, S.B., and Setlow, P. (2003). Mechanisms of killing of *Bacillus subtilis* spores by hypochlorite and chlorine dioxide. *J. Appl. Microbiol.* 95**,** 54–67.

10 Data Availability Statement

The datasets [GENERATED/ANALYZED] for this study can be found in the [NAME OF REPOSITORY] [LINK]. Please see the Data Availability section of the Author guidelines for more details.

11 Tables

Table 1. *B. subtilis* strains deficient in spore components used in this study.

Strain	*Genotype*	*Absent component(s) / Protection mechanism(s)*	*Reference*
PS832	Wild-type parental strain of PS and FB strains (prototroph; Trp^+ revertant of strain 168)	None / Wild-type / Full protection capabilities	(Popham *et al.*, 1995)
PY79	Wild-type parental strain of all PE strains (prototroph)	Wild-type / Full protection capabilities	(McKenney and Eichenberger, 2012)
PS283	Δ*sspA*	α-Type small, acid-soluble protein (SASP) / DNA protection	(Mason and Setlow, 1986)
PS338	Δ*sspB*	β-Type SASP / DNA protection	(Mason and Setlow, 1986)
PS483	Δ*sspE*	γ-Type SASP/ No protection function	(Hackett and Setlow, 1988)
PS356	Δ*sspA* Δ*sspB*	α- and β-Type SASP / DNA protection	(Loshon *et al.*, 1999)
PS482	Δ*sspA* Δ*sspB* Δ*sspE*	α-, β-, and γ-Type SASP / DNA protection	(Tovar-Rojo and Setlow, 1991)
PS1899	*dacB::cat*	Carboxypeptidase DacB / Core dehydration	(Popham *et al.*, 1995)
PS2211	*dacB::cat* Δ*sspA* Δ*sspB*	*dacB*, α/β-type SASP / Core dehydration and DNA protection	(Popham *et al.*, 1995)
PS3394	Δ*cotE;* Tet^R	CotE protein / Outer coat assembly	(Young and Setlow, 2003)
PE566	Δ*cotVW;* Erm^R	CotVW proteins / Spore crust assembly	(Eichenberger *et al.*, 2004)
PE620	Δ*cotX* Δ*cotYZ;* Neo^R	CotX and CotYZ proteins / Spore crust assembly	(McKenney and Eichenberger, 2012)
PE618	Δ*cotE;* Cat^R	CotE protein/ Outer coat assembly	(McKenney and Eichenberger, 2012)
PE277	Δ*safA;* Tet^R	SafA protein / Inner coat assembly	(McKenney and Eichenberger, 2012)
PE1720	Δ*cotE* Δ*safA;* Cat^R Tet^R	CotE and SafA proteins / Inner and outer coat assembly	(Raguse *et al.*, 2016)
PS3395	Δ*cotE* Δ*sspA* Δ*sspB;* Tet^R	CotE and α/β-type SASP / Outer coat assembly and DNA protection	(Young and Setlow, 2003)
FB122	Δ*sleB* Δ*spoVF;* Spc^R Tet^R	Enzymes SleB and dipicolinate synthase (SpoVF) / Degradation of the spore cortex in germination and DPA synthesis in the mother cell	(Magge *et al.*, 2008)
PS3664	Δ*sleB* Δ*spoVF* Δ*sspA* Δ*sspB;* Spc^R Tet^R	SleB and SpoVF, α/β-type SASP / DPA formation and DNA protection	(Setlow *et al.*, 2006)
PS3747	Δ*cotE::cam* Δ*sleB;* Spc^R Δ*spoVF* Δ*sspA* Δ*sspB;* Tet^R	*cotE*, DPA, α/β-type SASP / Outer coat assembly, DPA synthesis and DNA protection	(Setlow *et al.*, 2006)

Antibiotic resistance: Cat^R, resistance to chloramphenicol (5 μg mL^{-1}); Erm^R, resistance to erythromycin (2 μg mL^{-1}); Neo^R resistance to neomycin (10 μg mL^{-1}); Spc^R, resistant to spectinomycin (100 μg mL^{-1}); Tet^R, resistance to tetracycline (10 μg mL^{-1}).

Table 2. DNA repair-deficient *B. subtilis* strains used in this study.

Strain	*Genotype*	*Absent component / Repair mechanism(s)*	*Reference*
168	*trpC2*	Wild-type / Full DNA repair capabilities	Laboratory collection (Gunka *et al.*, 2012)
GP987	*trpC2 ΔdisA;* Tet^{R}	DNA integrity scanning protein DisA / Sporulation initiation	(Mehne *et al.*, 2013)
GP1503	*trpC2 ΔexoA::aphA3 Δnfo* Cat^{R}	Apurinic and apyrimidinic (AP) endonucleases ExoA and Nfo / Base excision repair pathway (BER)	(Gunka *et al.*, 2012)
BP141	*trpC2 ΔligD ku::aphA3*	Ku homodimer and DNA Ligase D / Non-Homologous End Joining (NHEJ)	This study
GP1167	*trpC2 Δmfd; Erm^{R}*	Transcription-repair coupling factor Mfd / Strand-specific DNA repair	(Gunka *et al.*, 2012)
GP1190	*trpC2 ΔmutSL::aphA3*	MutS and MutL proteins / Mismatch repair (MMR)	(Gunka *et al.*, 2012)
PERM715	*trpC2* pMUTIN4*::yqjH (polY1) ΔyqjW* (*polY2*); Em^{R} Kan^{R}	DNA polymerases Y1 and Y2 / Tranlesion synthesis (TLS)	(Rivas-Castillo *et al.*, 2010)
BP469	*trpC2 ΔrecA,* Erm^{R}	RecA protein / Homologous recombination (HR)	This study
GP894	*trpC2 ΔsbcDC::aphA3*	Exonuclease SbcDC / Inter-strand cross-link repair (ISCLR)	(Gunka *et al.*, 2012)
BP130	*trpC2 ΔsplB;* Spc^{r}	Spore photoproduct lyase (SP lyase) / SP repair	(Djouiai *et al.*, 2018)
RM1010	*trpC2 Δdis ΔsplB;* Tet^{R} Spc^{r}	SP lyase and DisA / SP repair and sporulation initiation	This study GP987 → BP130
RM1011	*trpC2 ΔexoA::aphA3 Δnfo ΔsplB;* Cat^{R} Spc^{R}	SP lyase, ExoA and Nfo / AP endonucleases and BER	This study GP1503 → BP130
RM1012	*trpC2 ΔligD ΔKu ΔsplB;* Spc^{R} Kan^{R}	SP lyase, Ku and LigD / SP repair, NHEJ	This study BP141 → BP130
RM1013	*trpC2 Δmfd ΔsplB;* Erm^{R} Spc^{R}	SP lyase and Mfd / SP repair and strand-specific DNA repair	This study GP1167 → BP130
RM1014	*trpC2 ΔmutSL::aphA3 ΔsplB;* Spc^{R}	SP lyase, MutS and MutL / SP repair and MMR	This study GP1190 → BP130
RM1015	*trpC2* pMUTIN4*::yqjH (polY1) ΔyqjW* (*polY2*) *ΔsplB;* Em^{R} Kan^{R} Spc^{R}	SP lyase, PolY1 and PolY2 / SP repair and TLS	This study WN1127 → BP130
RM1016	*trpC2 ΔsbcDC::aphA3*; Kan^{R} *ΔsplB*; Spc^{R}	SP lyase and exonuclease SbcDC / SP repair and ISCLR	This study GP894 → BP130
RM1017	*trpC2 ΔrecA ΔsplB;* Erm^{R} Spc^{R}	SP lyase and RecA / SP repair and HR	This study BP469 → BP130
GP1175	*trpC2 ΔuvrAB;* Erm^{R}	Excinuclease / Nucleotide Excision Repair (NER)	Gunka et al. (2012)
RM1019	*trpC2 ΔuvrAB;* Erm^{R} *ΔsplB;* Spc^{R}	SP lyase and UvrAB / SP repair and NER	This study GP1175 → BP130
PERM639	*ΔywjD::lacZ;* Erm^{R}	UV-damage-endonuclease (UVDE) / UV damage repair	(Ramirez-Guadiana *et al.*, 2012)
RM1021	*trpC2 ΔywjD::lacZ;* Erm^{R}	UVDE / UV damage repair	This study PERM639 → 168

RM1022	*trpC2* Δ*ywjD::lacZ;* Erm^{R} Δ*splB;* Spc^{R}	SP lyase and UVDE / SP repair and UV damage repair	This study PERM639 → BP130

[a] Arrows indicate constructions made by transformation.

Antibiotic resistance: Cat^{R}, resistance to chloramphenicol (5 µg mL^{-1}); Erm^{R}, resistance to erythromycin (2 µg mL^{-1}); *aphA3*: resistance to kanamycin (10 µg mL^{-1}); Spc^{R}, resistant to spectinomycin (100 µg mL^{-1}); Tet^{R}, resistance to tetracycline (10 µg mL^{-1}).

Table 3. Environmental conditions used during Mars environmental simulation experiments.

**Parameter**	**Value, fluence or percentage**
Pressure	0.69 ± 0.01 kPa
Temperature	-10 ± 2 °C
Relative humidity	8 ± 2 %
UV-VIS-NIR radiation[a]	Fluence rate per h (total applied fluence)[a]
Total UV (200-400 nm)	92.8 kJ m^{-2} h^{-1} (742.5 kJ/m^{-2})
UV-C (200-280 nm)	14.4 kJ m^{-2} h^{-1} (115.2 kJ/m^{-2})
UV-B (280-320 nm)	20.8 kJ m^{-2} h^{-1} (166.5 kJ/m^{-2})
UV-A (320-400 nm)	57.6 kJ m^{-2} h^{-1} (460.8 kJ/m^{-2})
VIS (400-700 nm)	864.0 kJ m^{-2} h^{-1} (6.91 MJ/m^{-2})
NIR (700-1100 nm)	882.0 kJ m^{-2} h^{-1} (7.05 MJ/m^{-2})
Total irradiance (200-1100 nm)	1838.8 kJ m^{-2} h^{-1} (14.7 MJ/m^{-2})
Time	24 h (with or without 8 h of radiation)
Mars Gas Mix[b]	95.54 % CO_2; 2.7% N_2, 1.6% Ar, 0.13% O_2, 0.03% H_2O

[a] Fluence rates for UVC and UVB were directly measured with an International Light, model IL400A radiometer (Newburyport, MA, USA).

[b] Fluence rates for UVA, total UV, VIS, and NIR were based on the models of (Schuerger *et al.*, 2005; Schuerger *et al.*, 2008).

[b] Gas composition in the MSC system was ordered from Boggs Gases, Inc. (Titusville, FL) as a commercial mixture of the top five gases in the Martian atmosphere (see Schuerger et al., 2008).

Table 4. Spore surviving fraction and increased sensitivity of mutant spores lacking protection mechanisms exposed to M(+)UV or M(-)UV.

	Surviving fraction		Increased sensitivity compared to wild-type spores (fold)	
**Protective component**	**M(+)UV**	**M(-)UV**	**M(+)UV**	**M(-)UV**
wild-type (wt, PS832)	(6.6 ± 0.8) × 10^{-2}	(7.3 ± 0.1) × 10^{-1}	1.0 ± 0.1	1.0 ± 0.2
sspA	(1.5 ± 0.2) × 10^{-2+}<br>[$^{+}$0.0042]	(2.0 ± 0.4) × 10^{-1+}<br>[$^{+}$0.0247]	4.4 ± 0.6^{+}<br>[$^{+}$0.0053]	3.6 ± 0.7^{+}<br>[$^{+}$0.0041]
sspB	(1.7 ± 0.2) × 10^{-2+}<br>[$^{+}$0.0058]	(4.6 ± 0.1) × 10^{-1+}<br>[$^{+}$0.0438]	3.8 ± 0.4^{+}<br>[$^{+}$0.0091]	1.6 ± 0.4<br>[0.1062]
sspE	(7.6 ± 0.1) × 10^{-2}<br>[0.2981]	(7.2 ± 0.1) × 10^{-1}<br>[0.8287]	0.9 ± 0.2<br>[0.4936]	1.0 ± 0.1<br>[0.5698]
sspA sspB	(2.4 ± 0.5) × 10^{-4+}<br>[$^{+}$0.0002]	(4.2 ± 0.9) × 10^{-2+}<br>[$^{+}$0.0025]	273 ± 57^{+}<br>[$^{+}$0.0015]	17 ± 3.8^{+}<br>[$^{+}$0.0021]
sspA sspB sspE	(1.5 ± 0.3) × 10^{-4+}<br>[$^{+}$0.0001]	(1.9 ± 0.4) × 10^{-2+}<br>[$^{+}$0.0016]	435 ± 36^{+}<br>[$^{+}$0.0012]	39 ± $9.3^{+\#}$<br>[$^{+}$0.0013; $^{\#}$0.0402]
dacB	(1.1 ± 0.2) × 10^{-2+}<br>[$^{+}$0.0032]	(1.5 ± 0.2) × 10^{-1+}<br>[$^{+}$0.0135]	6.1 ± 0.9^{+}<br>[$^{+}$0.0041]	4.8 ± 0.6^{+}<br>[$^{+}$0.0035]
dacB sspA sspB	(2.1 ± 0.2) × $10^{-5+\#}$<br>[$^{+}$0.0001; $^{\#}$0.0073]	(1.3 ± 0.2) × 10^{-2+}<br>[$^{+}$0.0011]	3172 ± $285^{+\#}$<br>[$^{+}$0.0001; $^{\#}$0.0086]	58 ± $9.6^{+\#}$<br>[$^{+}$0.0011; $^{\#}$0.0359]
sleB spoVF	(5.1 ± 0.2) × 10^{-2}<br>[0.0544]	(1.3 ± 0.2) × 10^{-1+}<br>[$^{+}$0.0109]	1.3 ± 0.2<br>[0.4628]	5.8 ± 1.0^{+}<br>[$^{+}$0.0031]
(*) sleB spoVF	(8.5 ± 0.7) × 10^{-2}<br>[0.0653]	(2.4 ± 0.5) × 10^{-1+}<br>[$^{+}$0.0214]	0.8 ± 0.2<br>[0.4897]	3.0 ± 0.6^{+}<br>[$^{+}$0.0068]
sleB spoVF sspA sspB	(1.8 ± 0.1) × $10^{-5+,\#}$<br>[$^{+}$0.0001; $^{\#}$0.0081]	(2.0 ± 0.3) × 10^{-3+}<br>[$^{+}$0.0001]	3780 ± $761^{+\#}$<br>[$^{+}$0.0001; $^{\#}$0.0063]	356 ± $57^{+\#}$<br>[$^{+}$0.0003; $^{\#}$0.0009]
(*) sleB spoVF sspA sspB	(1.7 ± 0.4) × 10^{-4+}<br>[$^{+}$0.0001]	(1.7 ± 0.2) × 10^{-2+}<br>[$^{+}$0.0009]	394 ± 66^{+}<br>[$^{+}$0.0012]	44 ± $6.2^{+\#}$<br>[$^{+}$0.0017; $^{\#}$0.0093]
cotE	(1.6 ± 0.3) × 10^{-2+}<br>[$^{+}$0.0083]	(8.4 ± 0.1) × 10^{-2+}<br>[$^{+}$0.0046]	4.2 ± 0.9^{+}<br>[$^{+}$0.0068]	8.6 ± 1.3^{+}<br>[$^{+}$0.0024]
cotE sspA sspB	(1.7 ± 0.3) × $10^{-5+,\#}$<br>[$^{+}$0.0001; $^{\#}$0.0038]	(6.9 ± 0.1) × 10^{-3+}<br>[$^{+}$0.0001]	3793 ± $691^{+\#}$<br>[$^{+}$0.0001; $^{\#}$0.0052]	106 ± $18^{+\#}$<br>[$^{+}$0.0009; $^{\#}$0.0029]
cotE sleB spoVF sspA sspB	(4.8 ± 0.3) × $10^{-7+,\#}$<br>[$^{+}$0.0001; $^{\#}$0.0001]	(6.8 ± 0.1) × 10^{-5+}<br>[$^{+}$0.0001]	137245 ± $32024^{+\#}$<br>[$^{+}$0.0001; $^{\#}$0.0001]	10635 ± $2162^{+\#}$<br>[$^{+}$0.0001; $^{\#}$0.0001]
(*) cotE sleB spoVF sspA sspB	(7.9 ± 0.1) × $10^{-6+,\#}$<br>[$^{+}$0.0001; $^{\#}$0.0030]	(1.1 ± 0.3) × 10^{-3+}<br>[$^{+}$0.0001]	8403 ± $2187^{+,\#}$<br>[$^{+}$0.0001; $^{\#}$0.0024]	646 ± $150^{+,\#}$<br>[$^{+}$0.0002; $^{\#}$0.0004]
wt (PY79)	(1.3 ± 0.2) × 10^{-1}	(8.3 ± 0.1) × 10^{-1}	1.0 ± 0.4	1.0 ± 0.2
cotVW	(7.5 ± 2.8) × 10^{-2}<br>[0.1634]	(9.3 ± 0.1) × 10^{-1}<br>[0.2648]	1.6 ± 0.1<br>[0.0653]	0.9 ± 0.1<br>[0.4819]
cotX cotYZ	(1.0 ± 0.8) × 10^{-1}	(6.9 ± 0.1) × 10^{-1}	1.3 ± 0.2	1.2 ± 0.2

	[$^{+}$0.5628]	[0.2297]	[0.3984]	[0.2987]
cotE	$(9.0 \pm 0.1) \times 10^{-3+}$<br>[$^{+}$0.0047]	$(4.7 \pm 0.1) \times 10^{-2+}$<br>[$^{+}$0.0089]	$15 \pm 2.4^{+}$<br>[$^{+}$0.0029]	$18 \pm 3.9^{+}$<br>[$^{+}$0.0072]
safA	$(5.5 \pm 0.7) \times 10^{-4+}$<br>[$^{+}$0.0003]	$(1.3 \pm 0.2) \times 10^{-2+}$<br>[$^{+}$0.0053]	$244 \pm 33^{+}$<br>[$^{+}$0.0009]	$63 \pm 10^{+}$<br>[$^{+}$0.0029]
cotE safA	$(1.3 \pm 0.3) \times 10^{-4+}$<br>[$^{+}$0.0001]	$(2.8 \pm 0.7) \times 10^{-3+}$<br>[$^{+}$0.0001]	$1060 \pm 237^{+}$<br>[$^{+}$0.0001]	$293 \pm 74^{+}$<br>[$^{+}$0.0015]

(*) DPA supplementation during sporulation.

$^{+}$ Statistically significant different from values for wild-type spores ($P \leq 0.05$); individual P values are given in brackets below the initial values.
$^{\#}$ Statistically significant difference between values for these mutant spores compared to values for *sspA sspB* spores ($P \leq 0.05$); individual P values are given in brackets below the initial values.

The surviving fraction was determined after a 24 h exposure to M(+/-)UV relative to that of control spores of each genotype, which were stored in air at room temperature (20 ± 2 °C), at relative humidity of 40 ± 5 % and protected from UV radiation. Increased sensitivity was determined relative to the respective wild-type spores as the ratio of the surviving fraction of wild-type over the surviving fraction of the various mutant spores. Three biological replicates were analyzed for each condition.

Table 5. Spore surviving fraction and increased sensitivity of mutant spores lacking DNA-repair proteins exposed to M(+UV) and M(-UV).

	**Survival fraction**		**Increased sensitivity compared to wild-type spores**		**Increased sensitivity compared to *splB* spores**	
**DNA repair**	**M(+)UV**	**M(-)UV**	**M(+)UV**	**M-UV**	**M(+)UV**	**M(-)UV**
wild-type (wt, 168)	$(3.6 \pm 0.7) \times 10^{-2}$	$(7.1 \pm 0.9) \times 10^{-1}$	1.0 ± 0.2	1.0 ± 0.1	n.a.	n.a.
disA	$(1.8 \pm 0.3) \times 10^{-2+}$<br>[$^{+}$0.0164]	$(5.3 \pm 0.6) \times 10^{-1}$<br>[0.0943]	$2.0 \pm 0.3^{+}$<br>[$^{+}$0.0439]	1.3 ± 0.2<br>[0.6844]	n.a.	n.a.
recA	$(4.1 \pm 1.0) \times 10^{-4+}$<br>[$^{+}$0.0009]	$(9.6 \pm 2.0) \times 10^{-3+}$<br>[$^{+}$0.0017]	$87 \pm 20^{+}$<br>[$^{+}$0.0037]	$74 \pm 14^{+}$<br>[$^{+}$0.0009]	n.a.	n.a.
ligD ku	$(1.0 \pm 0.1) \times 10^{-3+}$<br>[$^{+}$0.0025]	$(2.4 \pm 0.5) \times 10^{-2+}$<br>[$^{+}$0.0093]	$35 \pm 3.7^{+}$<br>[$^{+}$0.0049]	$29 \pm 6.3^{+}$<br>[$^{+}$0.0024]	n.a.	n.a.
sbcDC	$(8.4 \pm 1.0) \times 10^{-3+}$<br>[$^{+}$0.0103]	$(1.7 \pm 0.2) \times 10^{-1+}$<br>[$^{+}$0.0158]	$4.2 \pm 0.6^{+}$<br>[$^{+}$0.0108]	$4.3 \pm 0.6^{+}$<br>[$^{+}$0.0153]	n.a.	n.a.
exoA nfo	$(1.8 \pm 0.3) \times 10^{-3+}$<br>[$^{+}$0.0063]	$(4.7 \pm 0.7) \times 10^{-2+}$<br>[$^{+}$0.0065]	$20 \pm 2.8^{+}$<br>[$^{+}$0.0071]	$15 \pm 2.3^{+}$<br>[$^{+}$0.0085]	n.a.	n.a.
mutSL	$(1.5 \pm 0.3) \times 10^{-2+}$<br>[$^{+}$0.0132]	$(1.5 \pm 0.3) \times 10^{-1+}$<br>[$^{+}$0.0127]	$2.4 \pm 0.5^{+}$<br>[$^{+}$0.0264]	$4.6 \pm 0.9^{+}$<br>[$^{+}$0.0188]	n.a.	n.a.
polY1 polY2	$(1.7 \pm 0.3) \times 10^{-2+}$<br>[$^{+}$0.0139]	$(2.9 \pm 0.4) \times 10^{-1+}$<br>[$^{+}$0.0338]	$2.2 \pm 0.5^{+}$<br>[$^{+}$0.0289]	$2.4 \pm 0.3^{+}$<br>[$^{+}$0.0225]	n.a.	n.a.
mfd	$(4.3 \pm 0.7) \times 10^{-3+}$<br>[$^{+}$0.0061]	$(1.2 \pm 0.1) \times 10^{-1+}$<br>[$^{+}$0.0055]	$8.3 \pm 1.4^{+}$<br>[$^{+}$0.0042]	$5.8 \pm 0.6^{+}$<br>[$^{+}$0.0102]	n.a.	n.a.
uvrAB	$(1.1 \pm 0.2) \times 10^{-3+}$<br>[$^{+}$0.0025]	$(9.3 \pm 2.0) \times 10^{-2+}$<br>[$^{+}$0.0041]	$33 \pm 5.2^{+}$<br>[$^{+}$0.0018]	$7.6 \pm 1.5^{+}$<br>[$^{+}$0.0084]	n.a.	n.a.
ywjD	$(1.8 \pm 0.3) \times 10^{-2+}$<br>[$^{+}$0.0325]	$(8.3 \pm 1.0) \times 10^{-1}$<br>[0.2978]	$2.0 \pm 0.4^{+}$<br>[$^{+}$0.0323]	0.9 ± 0.1<br>[0.8744]	n.a.	n.a.
splB	$(1.2 \pm 0.2) \times 10^{-4+}$<br>[$^{+}$0.0006]	$(4.1 \pm 0.5) \times 10^{-1}$<br>[0.0538]	$304 \pm 51^{+}$<br>[$^{+}$0.0004]	$1.7 \pm 0.2^{+}$<br>[$^{+}$0.0308]	1.0 ± 0.2	1.0 ± 0.2
disA splB	$(1.4 \pm 0.3) \times 10^{4+}$<br>[$^{+}$0.0005; 0.3901]	$(1.6 \pm 0.3) \times 10^{-1+\#}$<br>[$^{+}$0.0147; $^{\#}$0.0371]	$264 \pm 56^{+}$<br>[$^{+}$0.0009]	$4.5 \pm 1.0^{+}$<br>[$^{+}$0.0069]	0.9 ± 0.2<br>[0.8551]	$2.6 \pm 0.6^{\#}$<br>[$^{\#}$0.0319]
recA splB	$(1.2 \pm 0.2) \times 10^{5+\#}$<br>[$^{+}$0.0001; $^{\#}$0.0044]	$(2.7 \pm 0.4) \times 10^{-3+\#}$<br>[$^{+}$0.0009; $^{\#}$0.0021]	$3089 \pm 501^{+}$<br>[$^{+}$0.0001]	$266 \pm 41^{+}$<br>[$^{+}$0.0007]	$10 \pm 1.6^{\#}$<br>[$^{\#}$0.0157]	$154 \pm 24^{\#}$<br>[$^{\#}$0.0007]
ligD ku splB	$(4.9 \pm 0.7) \times 10^{6+\#}$<br>[$^{+}$0.0001; $^{\#}$0.0006]	$(1.9 \pm 0.2) \times 10^{-2+\#}$<br>[$^{+}$0.0076; $^{\#}$0.0268]	$7285 \pm 1110^{+}$<br>[$^{+}$0.0001]	$38 \pm 4.9^{+}$<br>[$^{+}$0.0024]	$24 \pm 3.6^{\#}$<br>[$^{\#}$0.0046]	$22 \pm 2.9^{\#}$<br>[$^{\#}$0.0053]
sbcDC splB	$(2.3 \pm 0.3) \times 10^{5+\#}$<br>[$^{+}$0.0001; $^{\#}$0.0065]	$(1.7 \pm 0.3) \times 10^{-1+\#}$<br>[$^{+}$0.0139; $^{\#}$0.0427]	$1568 \pm 192^{+}$<br>[$^{+}$0.0001]	$4.1 \pm 0.7^{+}$<br>[$^{+}$0.0127]	$5.2 \pm 0.6^{\#}$<br>[$^{\#}$0.0226]	$2.4 \pm 0.4^{\#}$<br>[$^{\#}$0.0352]
exoA nfo splB	$(1.9 \pm 0.4) \times 10^{5+\#}$<br>[$^{+}$0.0001; $^{\#}$0.0038]	$(2.9 \pm 0.4) \times 10^{-2+\#}$<br>[$^{+}$0.0085; $^{\#}$0.0326]	$1895 \pm 399^{+}$<br>[$^{+}$0.0001]	$25 \pm 3.4^{+}$<br>[$^{+}$0.0055]	$6.2 \pm 1.3^{\#}$<br>[$^{\#}$0.0185]	$14 \pm 1.9^{\#}$<br>[$^{\#}$0.0078]
mutSL splB	$(1.1 \pm 0.2) \times 10^{4+}$<br>[$^{+}$0.0001; 0.5734]	$(3.9 \pm 0.6) \times 10^{-2+,\#}$<br>[$^{+}$0.0092; $^{\#}$0.0378]	$316 \pm 60^{+}$<br>[$^{+}$0.0005]	$18 \pm 2.9^{+}$<br>[$^{+}$0.0087]	1.0 ± 0.2<br>[0.9258]	$10 \pm 1.7^{\#}$<br>[$^{\#}$0.0105]
polY1,2 splB	$(6.3 \pm 1.0) \times 10^{5+\#}$	$(2.6 \pm 0.5) \times 10^{-1+}$	$568 \pm 86.5^{+}$	$2.7 \pm 0.5^{+}$	$1.9 \pm 0.3^{\#}$	1.6 ± 0.3

	[$^{+}$0.0001; $^{\#}$0.0125]	[$^{+}$0.0341; 0.0537]	[$^{+}$0.0003]	[$^{+}$0.0265]	[$^{\#}$0.0435]	[0.2495]
mfd splB	$(1.8 \pm 0.4) \times 10^{6+\#}$	$(6.2 \pm 1.0) \times 10^{-2,\#}$	$20092 \pm 4969^{+}$	$11 \pm 2.3^{+}$	$66 \pm 16^{\#}$	$6.6 \pm 1.3^{\#}$
	[$^{+}$0.0001; $^{\#}$0.0005]	[$^{+}$0.0032; $^{\#}$0.0043]	[$^{+}$0.0001]	[$^{+}$0.0105]	[$^{\#}$0.0012]	[$^{\#}$0.0194]
uvrAB splB	$(4.6 \pm 0.7) \times 10^{7+\#}$	$(5.0 \pm 0.7) \times 10^{-2+\#}$	$77260 \pm 12195^{+}$	$14 \pm 1.9^{+}$	$254 \pm 40^{\#}$	$8.3 \pm 1.1^{\#}$
	[$^{+}$0.0001; $^{\#}$0.0001]	[$^{+}$0.0065; $^{\#}$0.0025]	[$^{+}$0.0001]	[$^{+}$0.0096]	[$^{\#}$0.0007]	[$^{\#}$0.0183]
ywjD splB	$(1.8 \pm 0.3) \times 10^{5+\#}$	$(7.3 \pm 0.1) \times 10^{-1}$	$1958 \pm 357^{+}$	1.0 ± 0.2	$6.4 \pm 1.2^{\#}$	0.7 ± 0.2
	[$^{+}$0.0001; $^{\#}$0.0036]	[0.7152; $^{\#}$0.1986]	[$^{+}$0.0001]	[0.8749]	[$^{\#}$0.0175]	[0.7541]

n.a. = not applicable

$^{+}$ Statistically significant difference from values for wild-type spores ($P \leq 0.05$); individual P values are given in brackets below the initial values.

$^{\#}$ Statistically significant difference between values for these mutant spores compared to values for *splB* spores ($P \leq 0.05$); individual P values are given in brackets below the initial values.

The surviving fraction was determined after 24 h exposure to M(+/-)UV relative to that of control spores of each genotype, which were stored in air at room temperature (20 ± 2 °C), at relative humidity of 40 ± 5 % and protected from UV radiation. Increased sensitivity was determined relative to the respective wild-type or *splB* spores as the ratio of the surviving fraction of wild-type or *splB* spores over the surviving fraction of the various mutant spore. Three biological replicates were analyzed for each condition.

12 Figure legends

Figure 1. *Bacillus subtilis* spore structure depicting the main resistance mechanisms analyzed in the current study. Each protection (in yellow) or DNA repair (in green) mechanism is represented by a symbol. Each symbol is coupled with a small description of the gene that is mutated, the protein it codes for, followed by the main cellular event it is involved in. The location of the symbol corresponds to the main place of action within the spore. More information on the mechanisms of DNA protection, repair, dehydration, and coat assembly is provided in the introduction.

Figure 2. Sporulation deficiency (in %) of *B. subtilis* spores deficient in protection mechanisms exposed to simulated Martian conditions, measured as Spo- colonies per 250 colonies of survivors of DNA repair deficient spores exposed to M(+)UV (white bars) or M(-)UV (grey bars). (*) depicts significance after paired t-test $P < 0.05$, when compared with the respective wild-type. Data are expressed as averages and standard deviations.

Figure 3. Sporulation deficiency (in %) of *B. subtilis* spores deficient in DNA repair mechanisms exposed to simulated Martian conditions, measured as Spo- colonies per 250 colonies of survivors of DNA repair deficient spores exposed to M(+)UV (white bars) or M(-)UV (grey bars). (*) depicts significance after paired t-test $P < 0.05$, when compared with the respective wild-type. Data are expressed as averages and standard deviations.

Figure 4. Major factors involved in *B. subtilis* spore resistance to simulated Mars surface conditions. The main mutant genotypes (left) and missing mechanisms of protection or repair (right) are presented, providing a comparison between *B. subtilis* spore sensitivity after exposure to the M(-)UV and M(+)UV Martian environments (see Tables 4 and 5 for information on all tested genotypes). Fold sensitivity was calculated as "mutant versus wild-type" measuring spore survival by colony formation. Different fold-sensitivity values are represented in a color code from <10 to $<10^6$-fold, all comparing sensitivities of wild-type and mutant spores as determined in Tables 4 and 5.
