# Supplementary figures and images for: Bacillus subtilis Spore Resistance to Simulated Mars Surface Conditions
Source: Front Microbiol. 2019 Feb 26;10:333. doi: 10.3389/fmicb.2019.00333 (PMC6399134; doi:10.3389/fmicb.2019.00333)

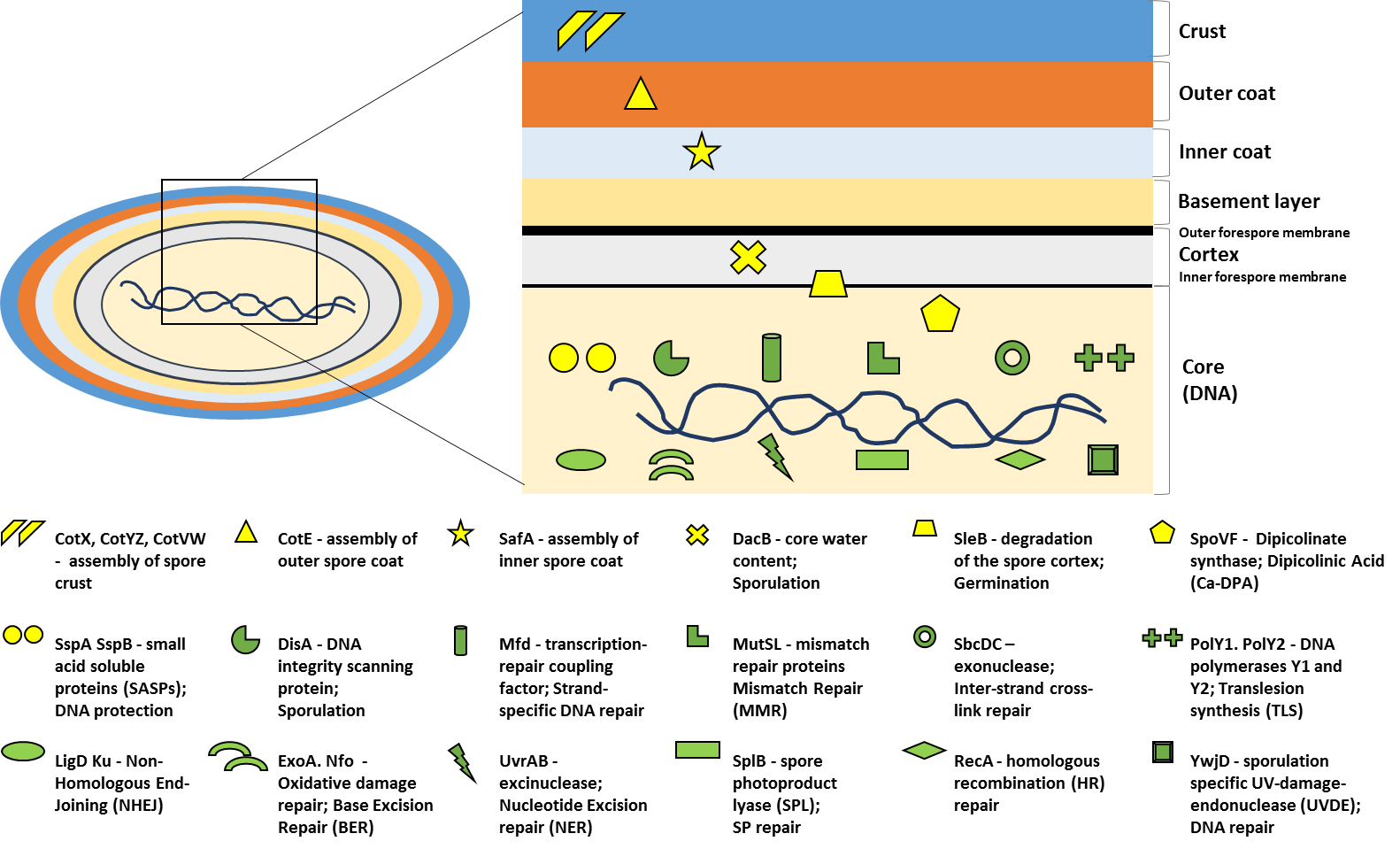

Supplement: Supplementary file 2 [file Data_Sheet_2.ZIP › Figure_1_Cortesao et al_Bacillus subtilis spore resistance to simulated Mars surface conditions.tif]

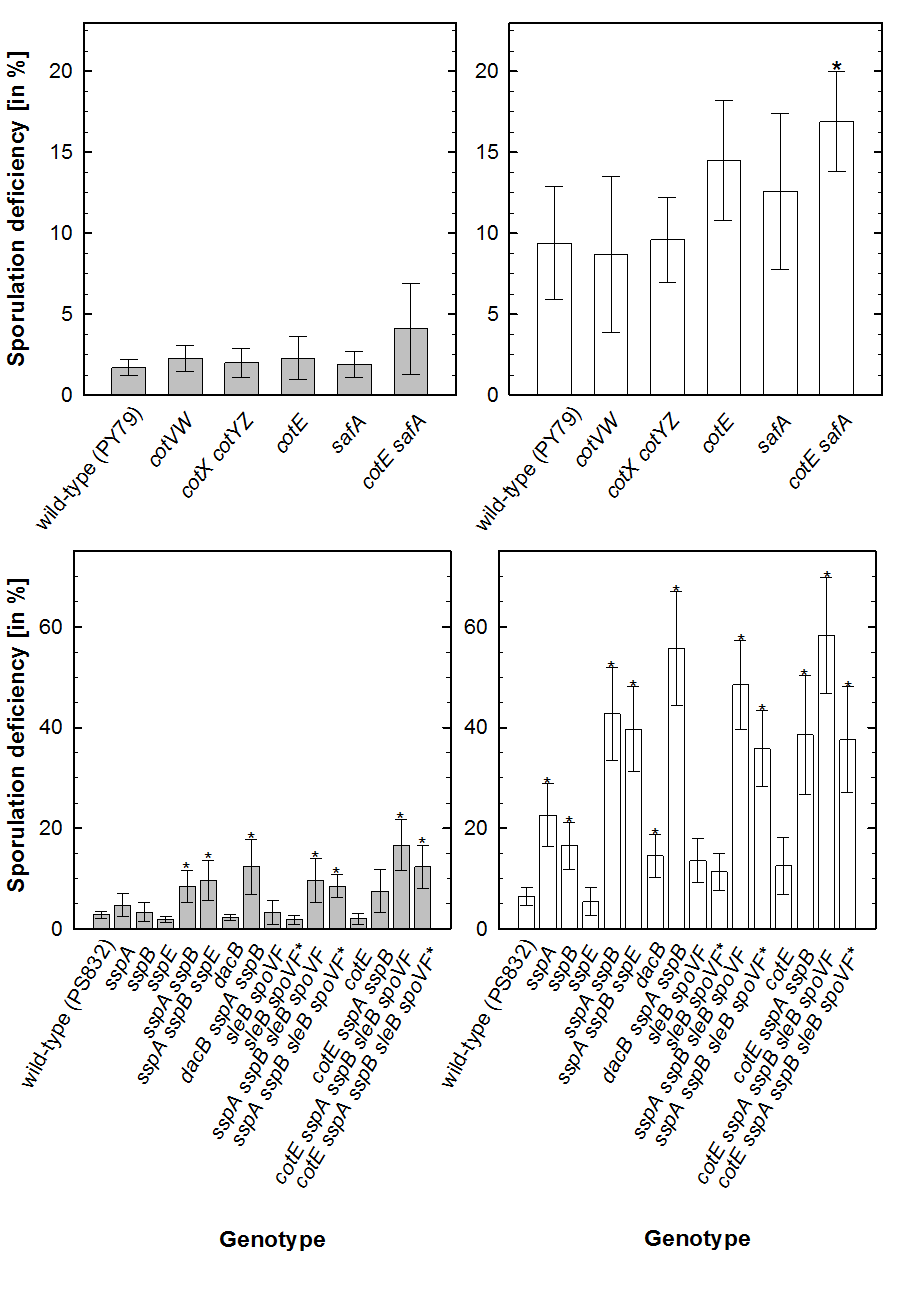

Supplement: Supplementary file 2 [file Data_Sheet_2.ZIP › Figure_2_Cortesao et al_Bacillus subtilis spore resistance to simulated Mars surface conditions.tif]

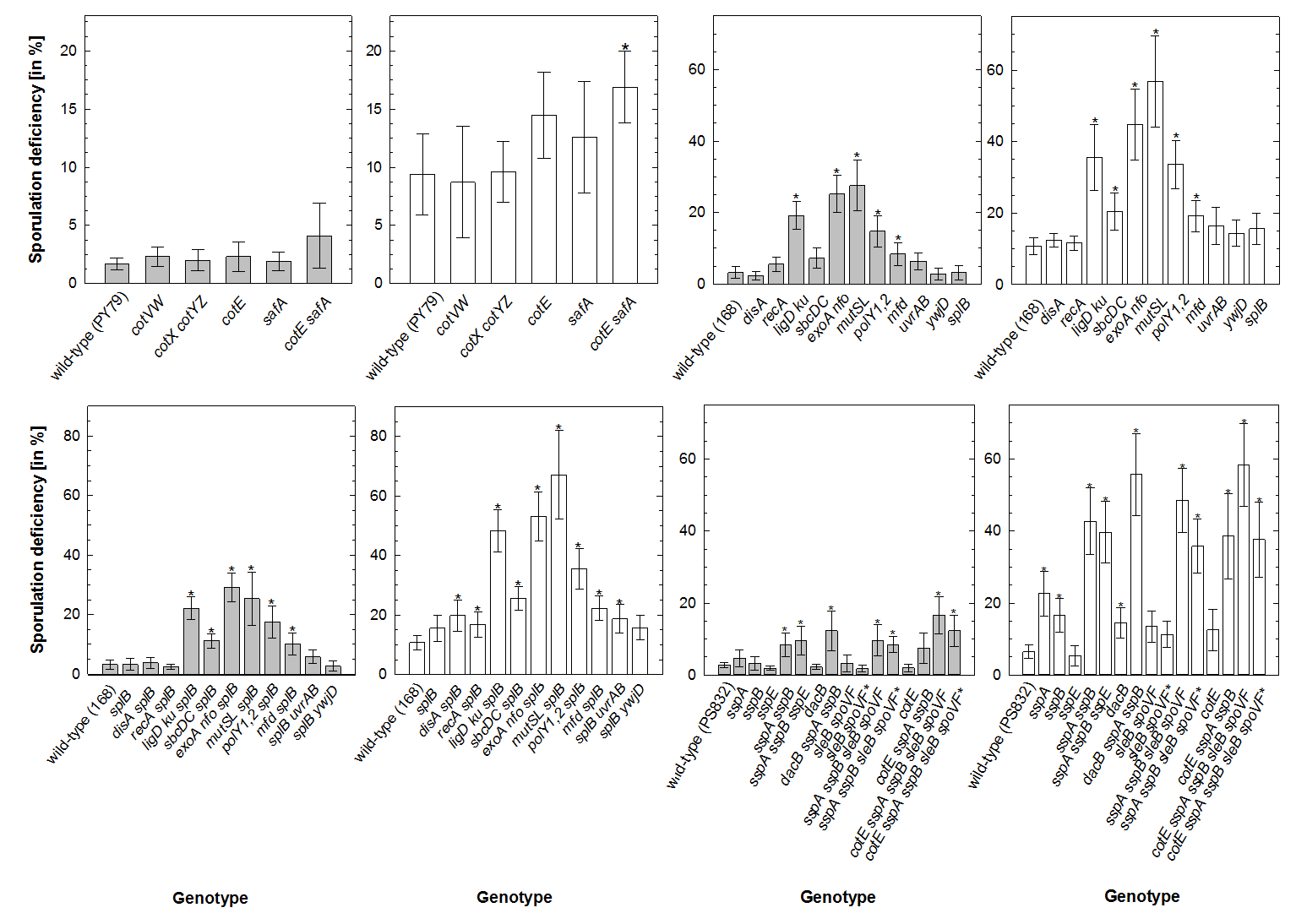

Supplement: Supplementary file 2 [file Data_Sheet_2.ZIP › Figure_2and3_Cortesao et al_Bacillus subtilis spore resistance to simulated Mars surface conditions.tif]

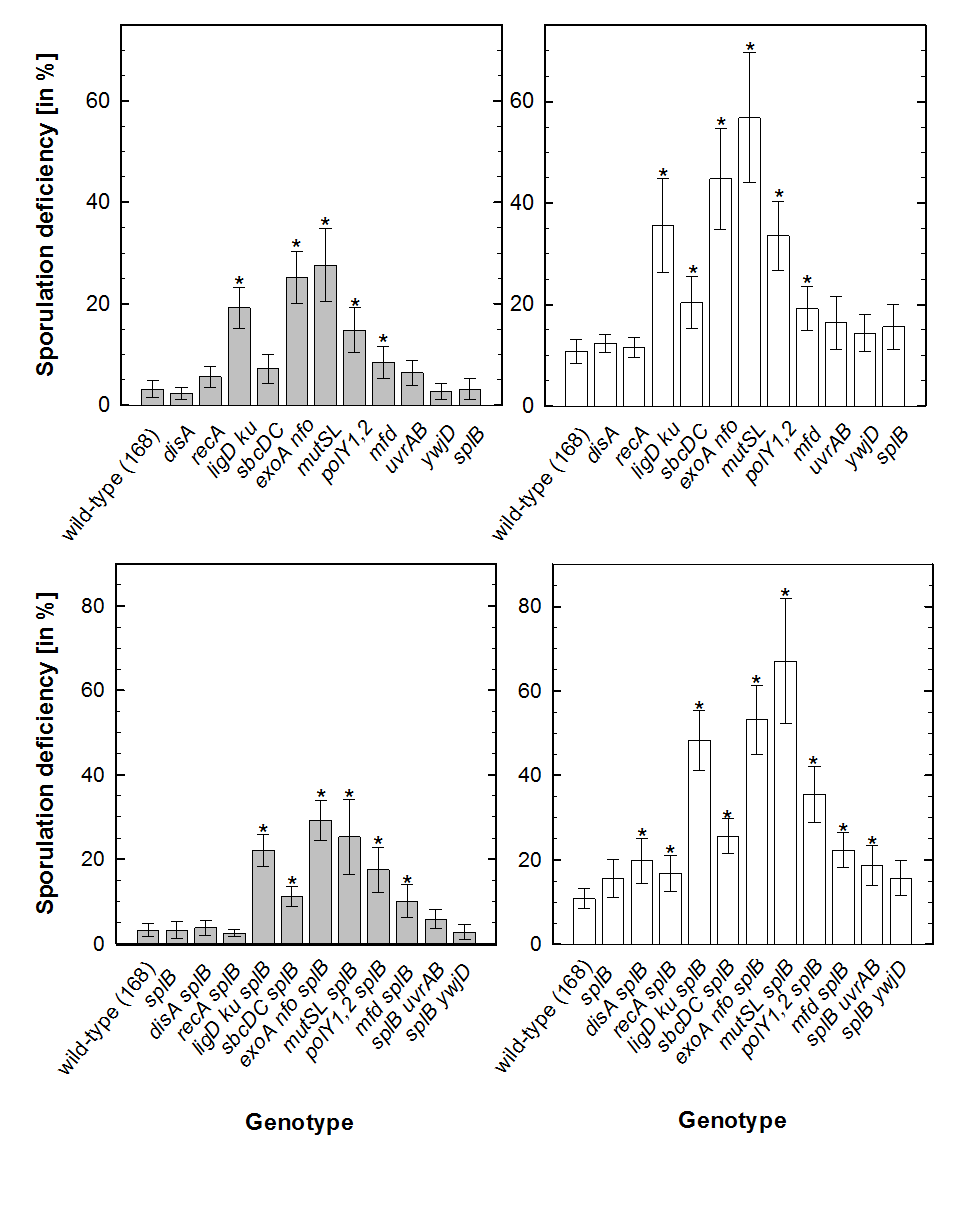

Supplement: Supplementary file 2 [file Data_Sheet_2.ZIP › Figure_3_Cortesao et al_Bacillus subtilis spore resistance to simulated Mars surface conditions.tif]

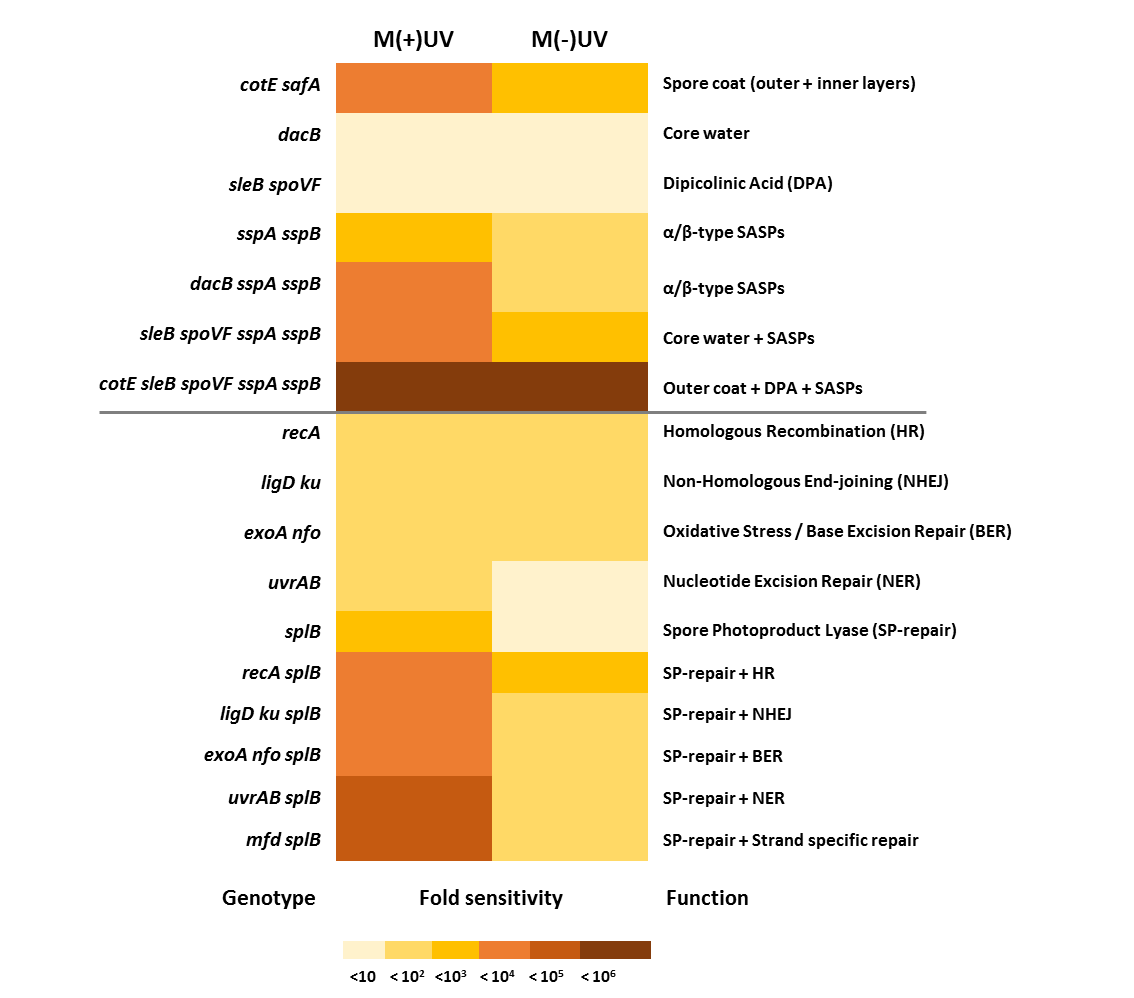

Supplement: Supplementary file 2 [file Data_Sheet_2.ZIP › Figure_4_Cortesao et al_Bacillus subtilis spore resistance to simulated Mars surface conditions.tif]

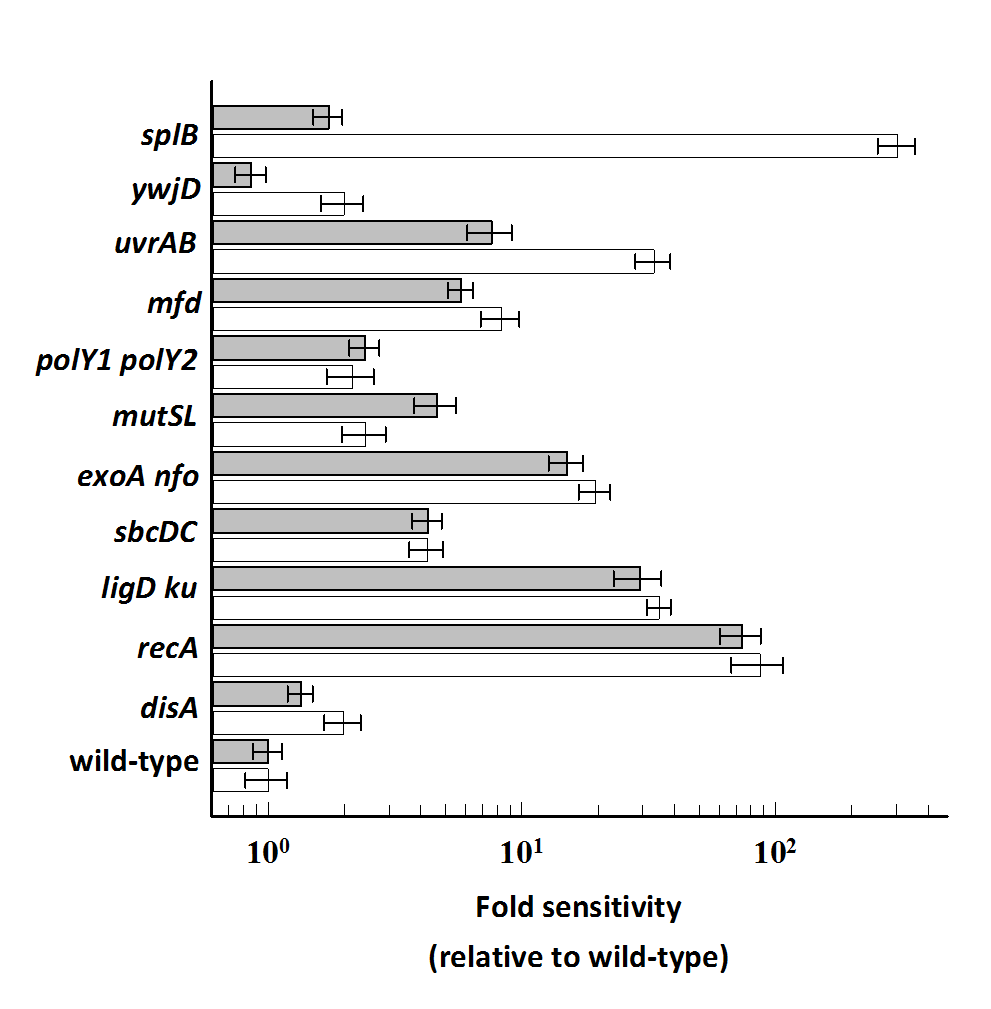

Supplement: Supplementary file 2 [file Data_Sheet_2.ZIP › Supp_10_Cortesao et al_Bacillus subtilis spore resistance to simulated Mars surface conditions.tif]

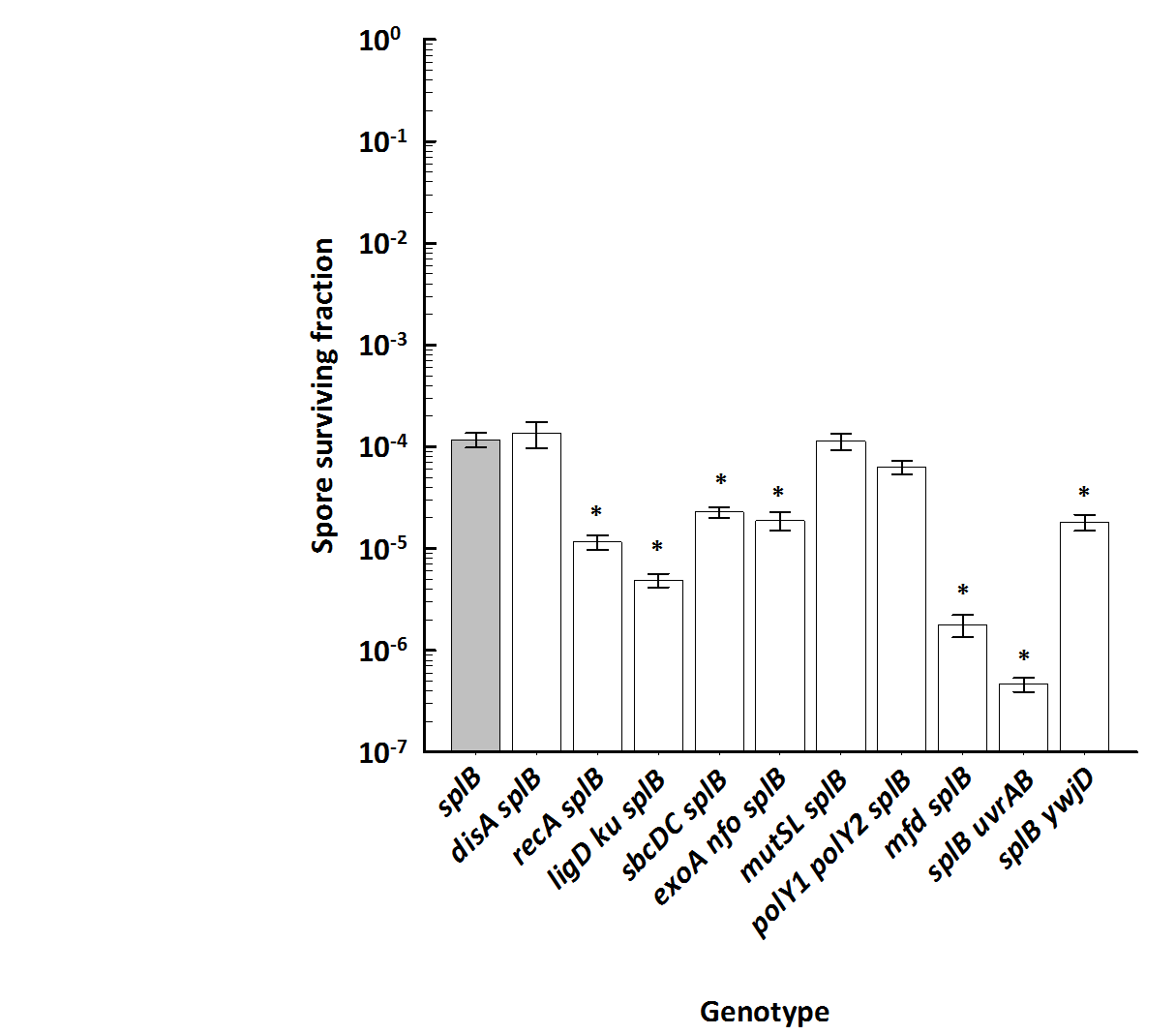

Supplement: Supplementary file 2 [file Data_Sheet_2.ZIP › Supp_11_Cortesao et al_Bacillus subtilis spore resistance to simulated Mars surface conditions.tif]

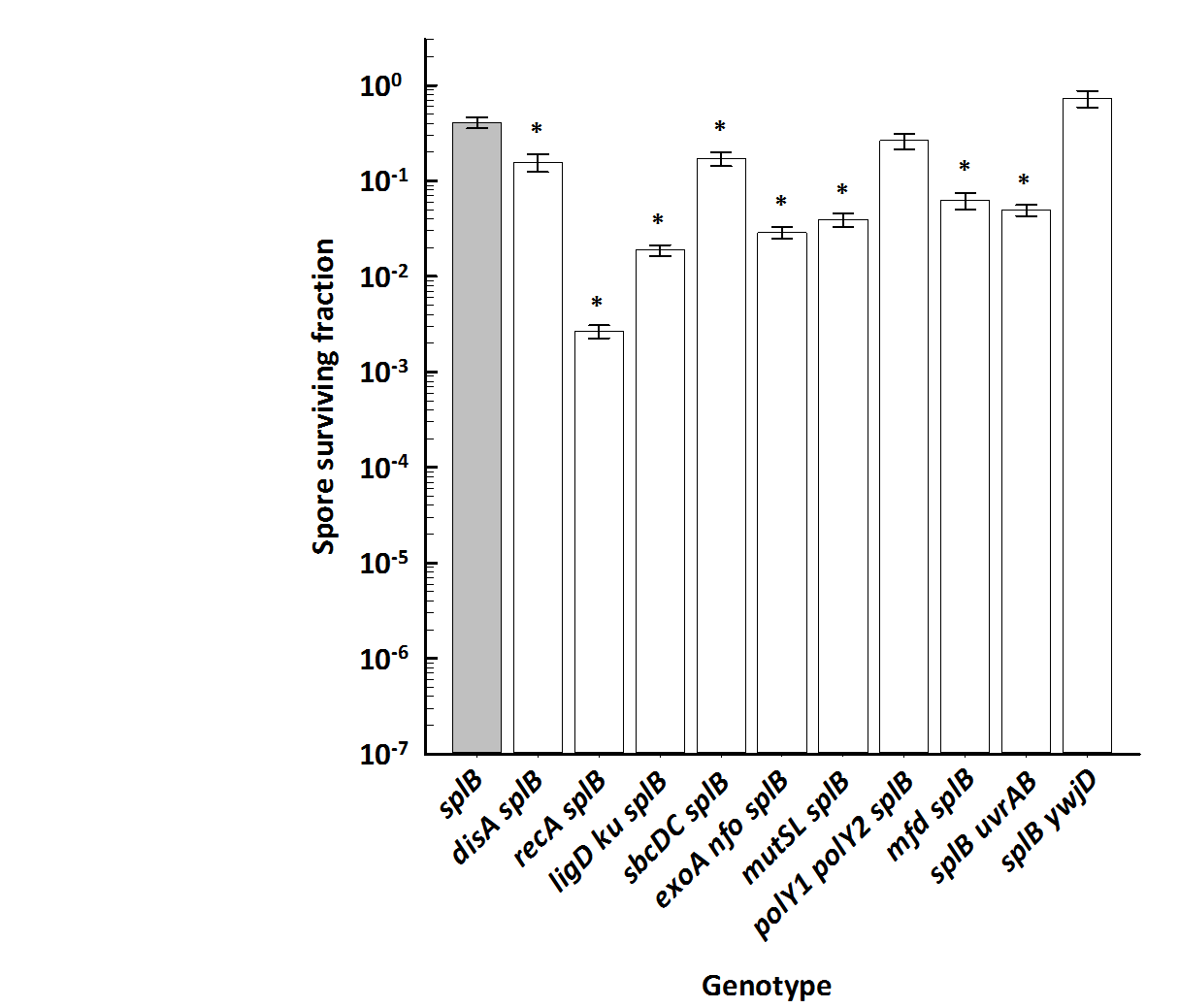

Supplement: Supplementary file 2 [file Data_Sheet_2.ZIP › Supp_12_Cortesao et al_Bacillus subtilis spore resistance to simulated Mars surface conditions.tif]

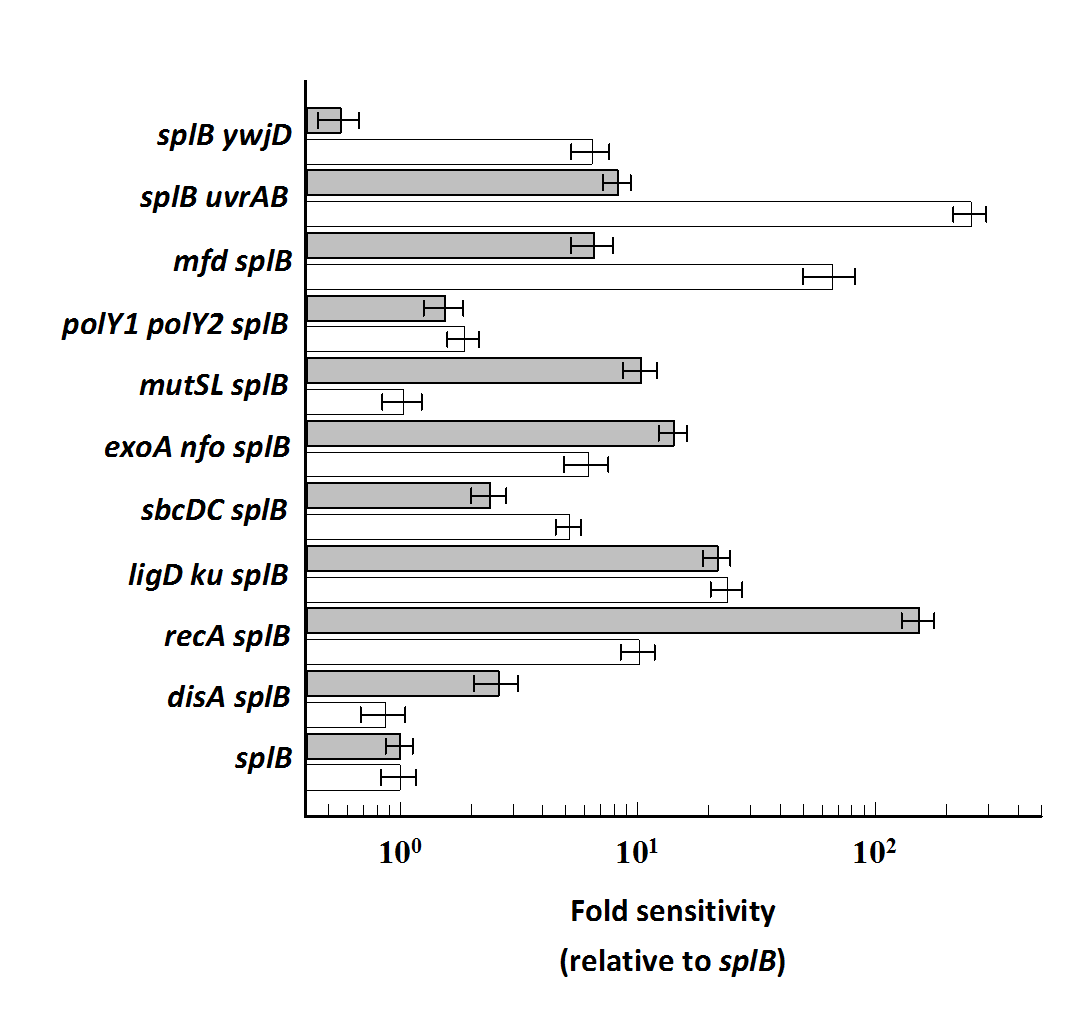

Supplement: Supplementary file 2 [file Data_Sheet_2.ZIP › Supp_13_Cortesao et al_Bacillus subtilis spore resistance to simulated Mars surface conditions.tif]

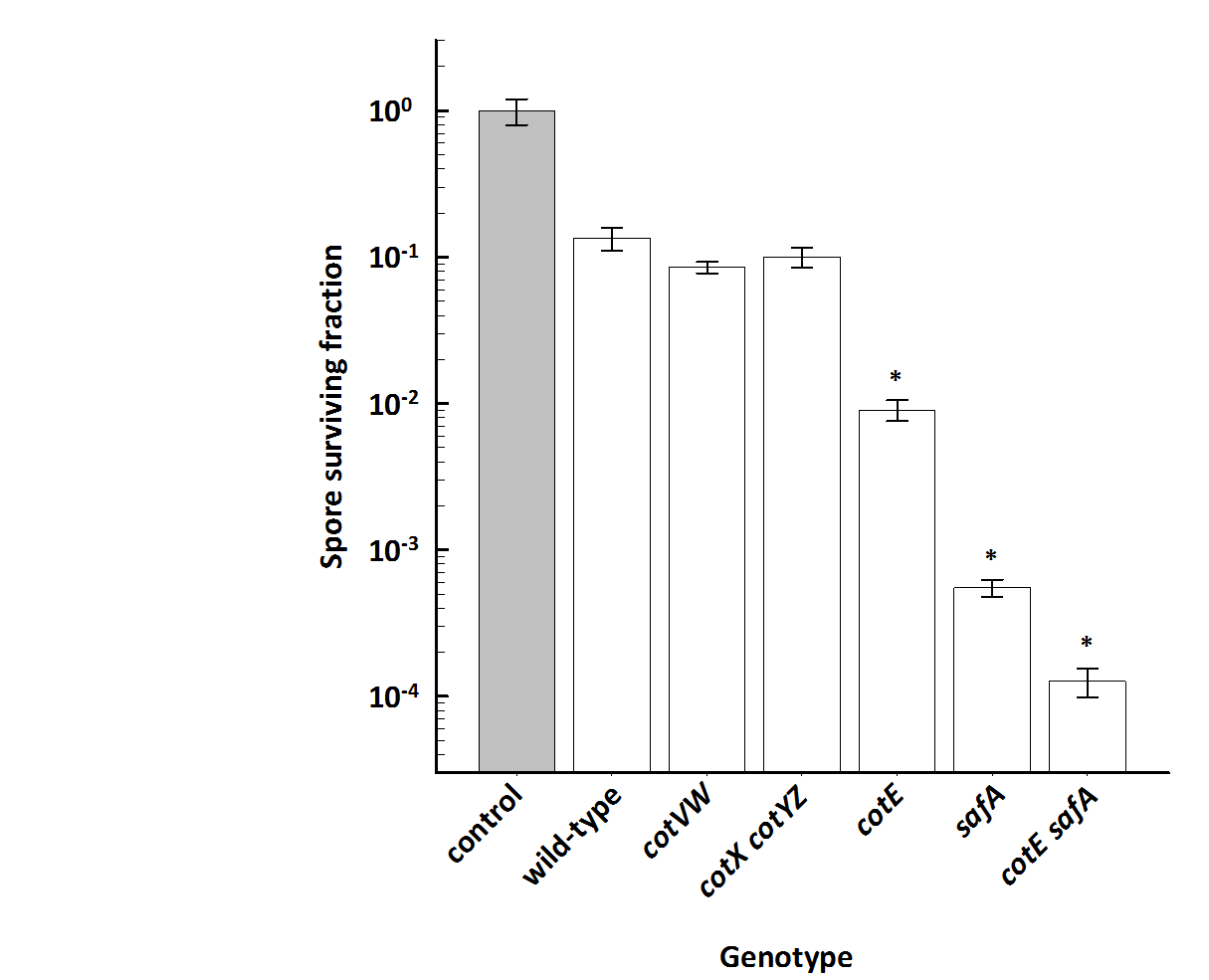

Supplement: Supplementary file 2 [file Data_Sheet_2.ZIP › Supp_1_Cortesao et al_Bacillus subtilis spore resistance to simulated Mars surface conditions.tif]

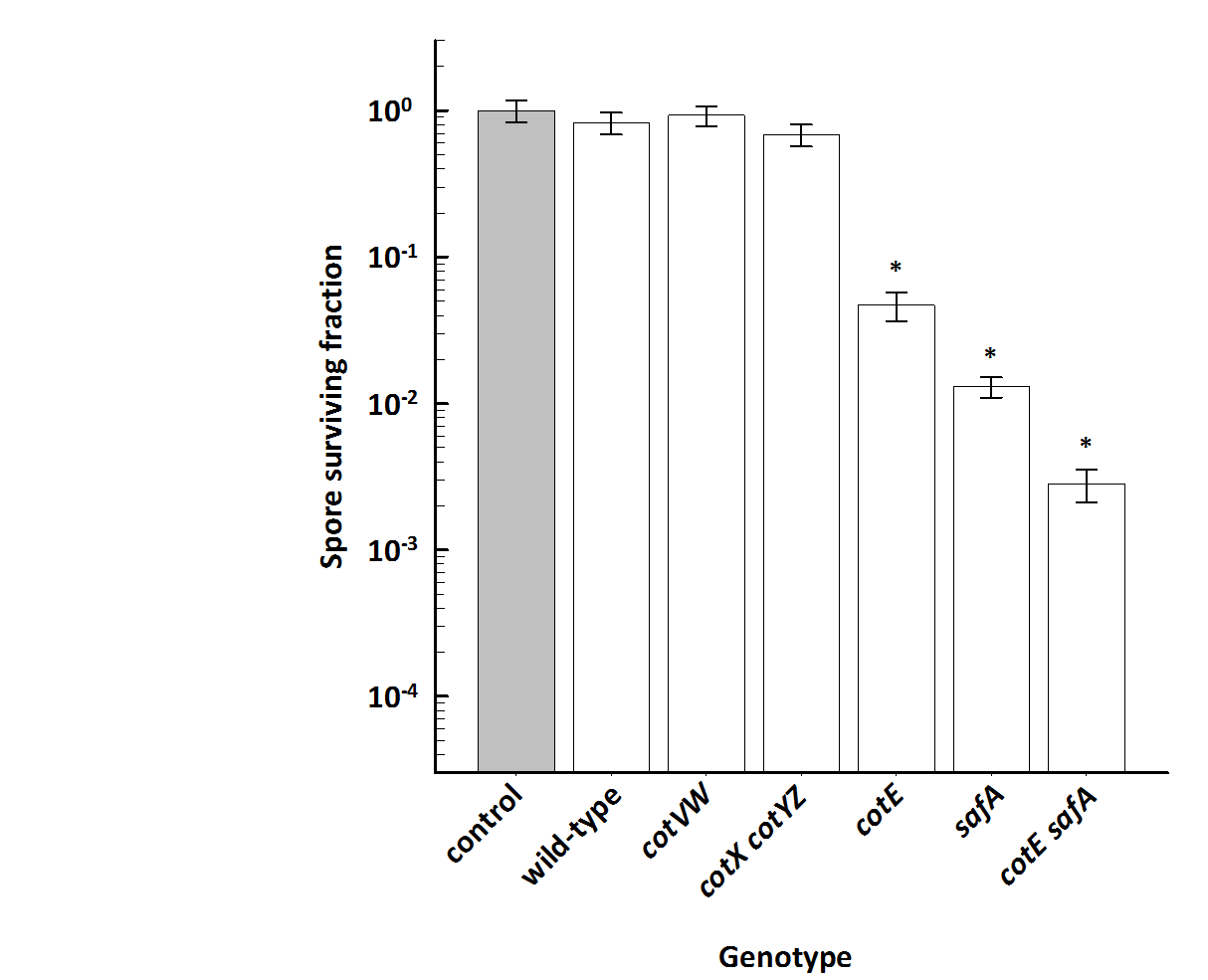

Supplement: Supplementary file 2 [file Data_Sheet_2.ZIP › Supp_2_Cortesao et al_Bacillus subtilis spore resistance to simulated Mars surface conditions.tif]

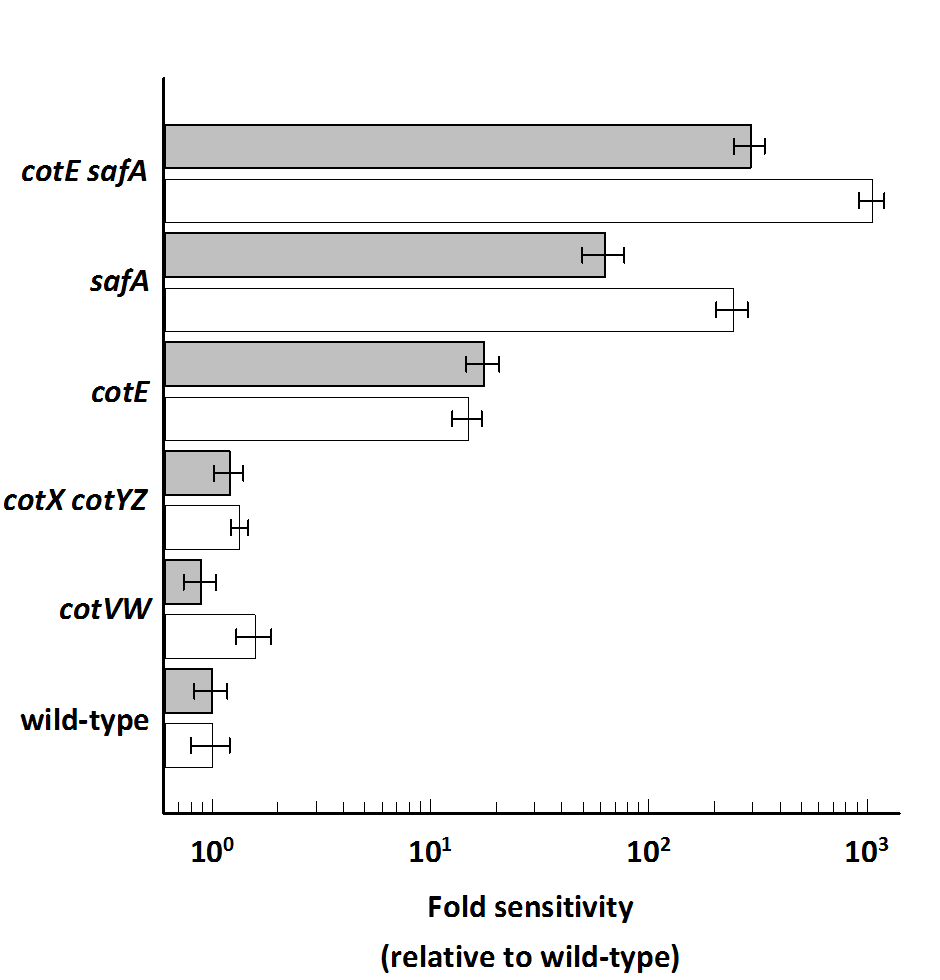

Supplement: Supplementary file 2 [file Data_Sheet_2.ZIP › Supp_3_Cortesao et al_Bacillus subtilis spore resistance to simulated Mars surface conditions.tif]

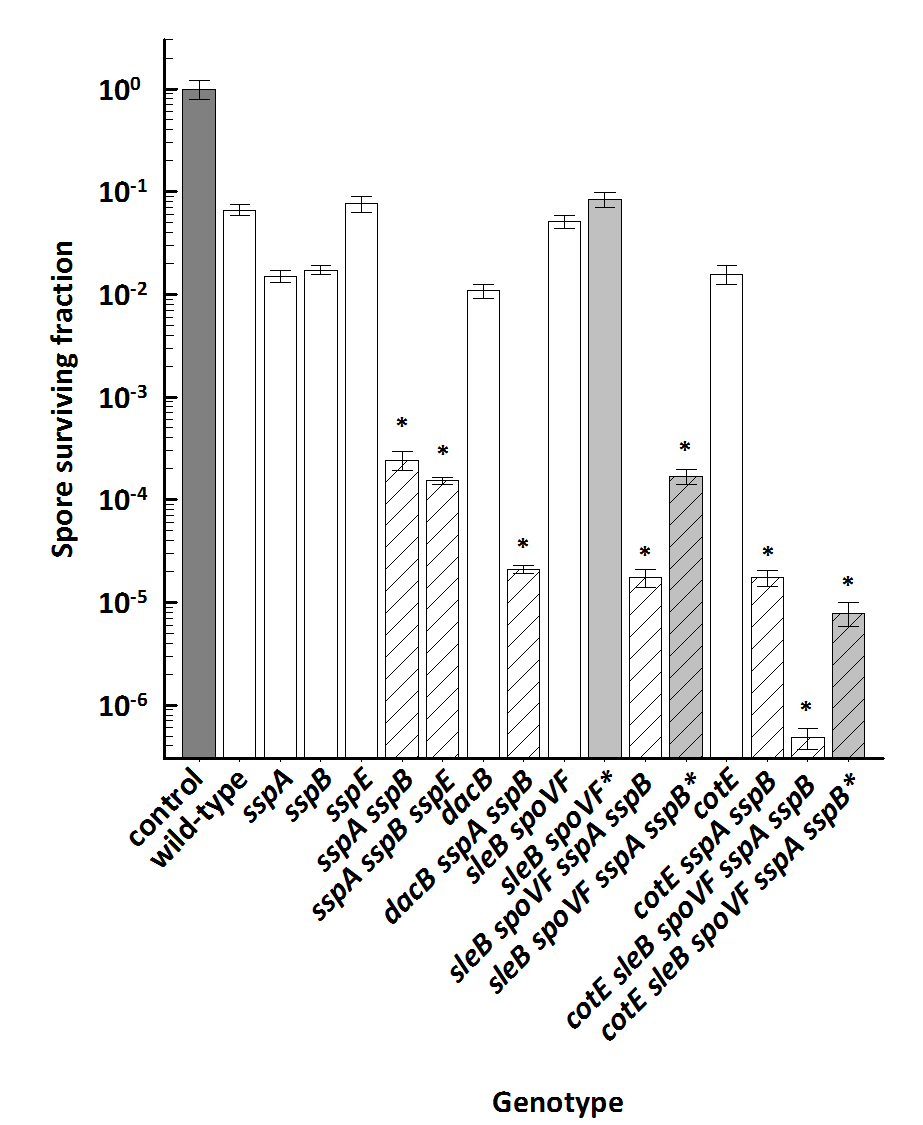

Supplement: Supplementary file 2 [file Data_Sheet_2.ZIP › Supp_4_Cortesao et al_Bacillus subtilis spore resistance to simulated Mars surface conditions.tif]

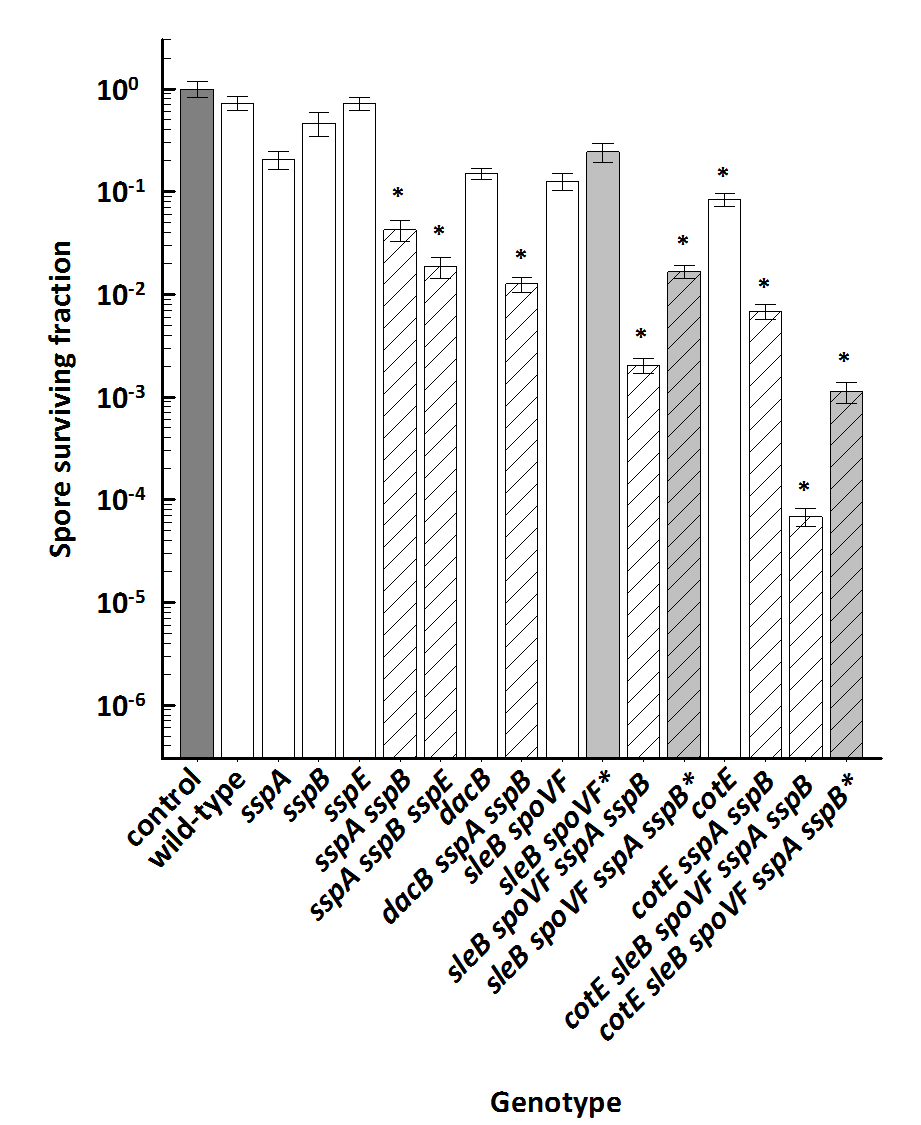

Supplement: Supplementary file 2 [file Data_Sheet_2.ZIP › Supp_5_Cortesao et al_Bacillus subtilis spore resistance to simulated Mars surface conditions.tif]

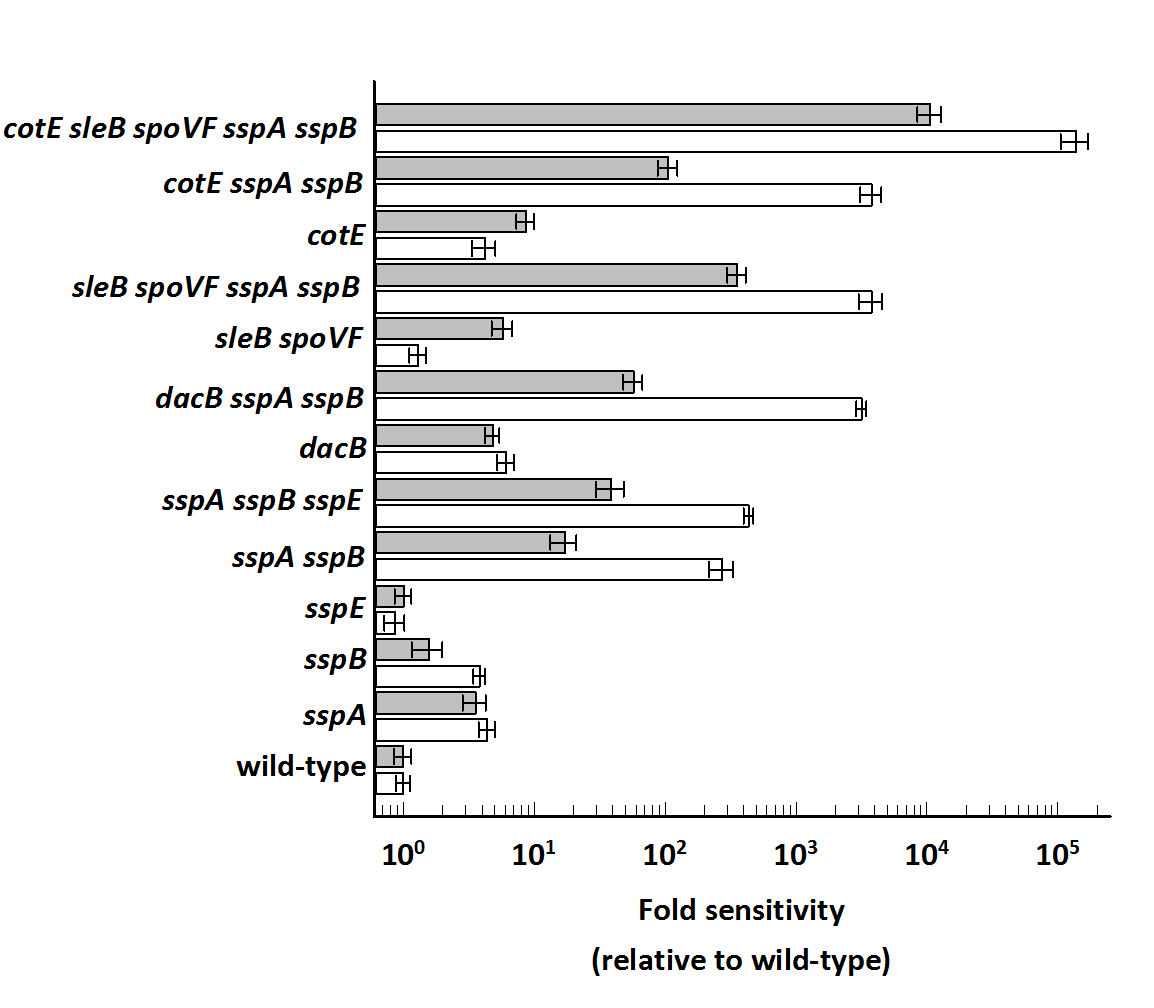

Supplement: Supplementary file 2 [file Data_Sheet_2.ZIP › Supp_6_Cortesao et al_Bacillus subtilis spore resistance to simulated Mars surface conditions.tif]

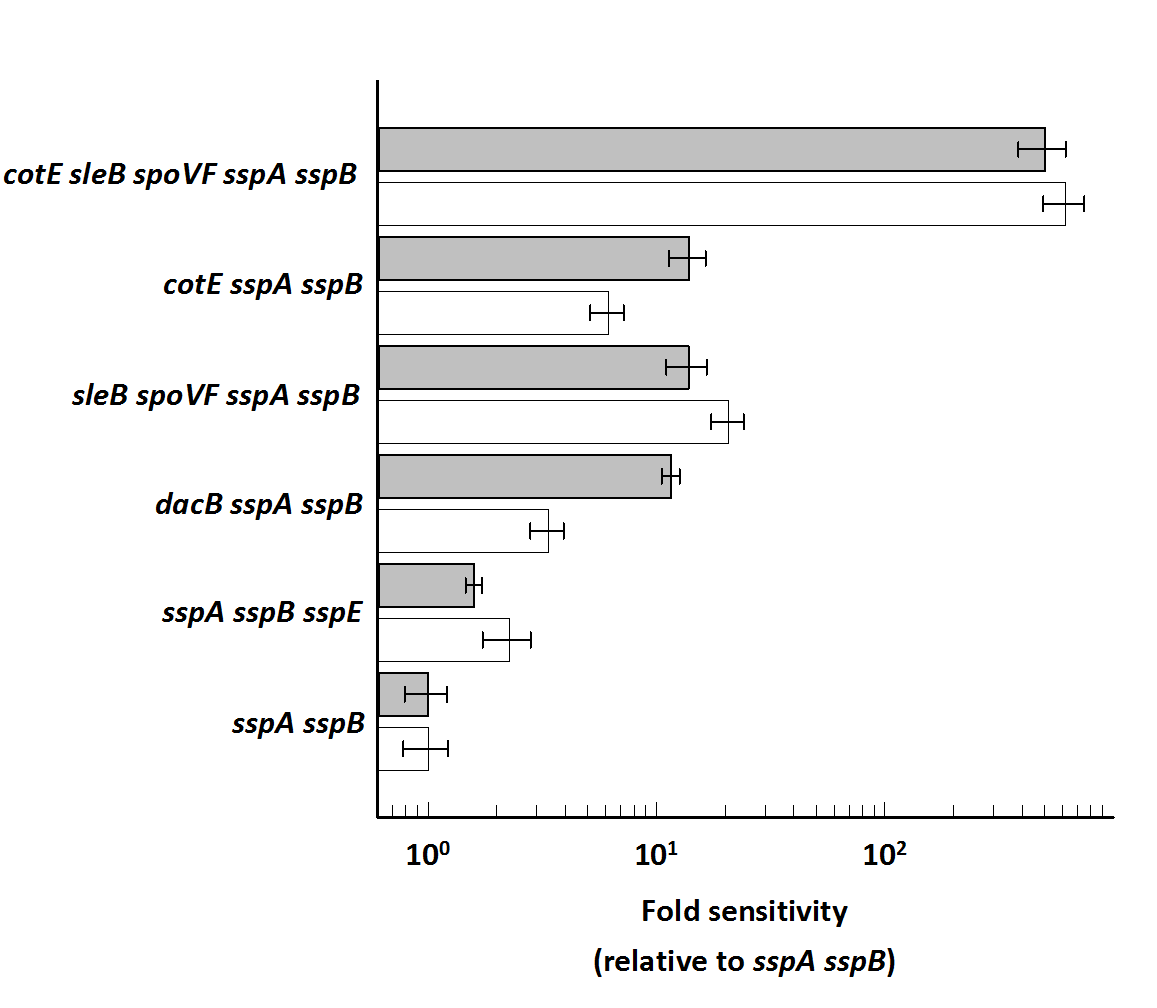

Supplement: Supplementary file 2 [file Data_Sheet_2.ZIP › Supp_7_Cortesao et al_Bacillus subtilis spore resistance to simulated Mars surface conditions.tif]

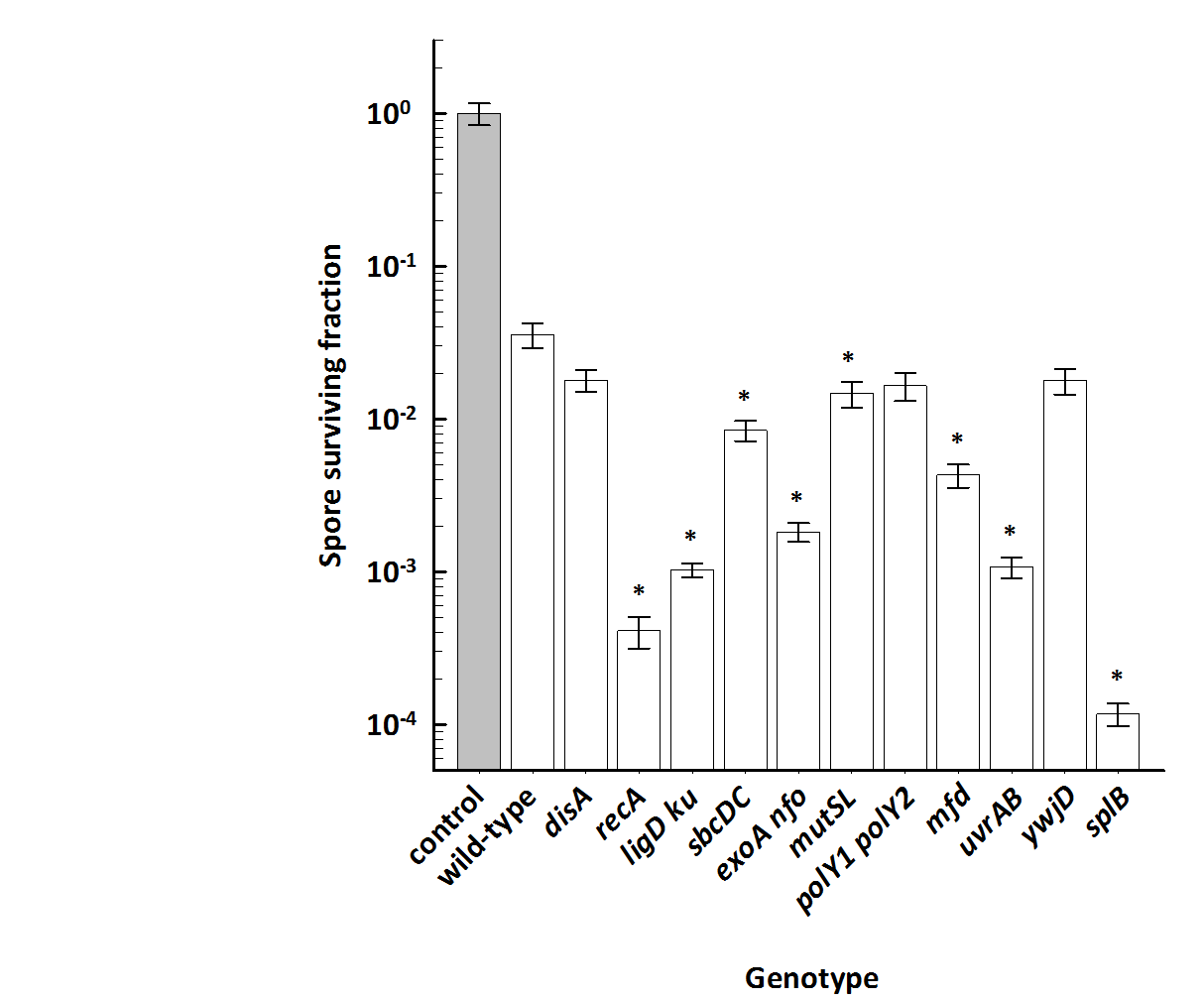

Supplement: Supplementary file 2 [file Data_Sheet_2.ZIP › Supp_8_Cortesao et al_Bacillus subtilis spore resistance to simulated Mars surface conditions.tif]

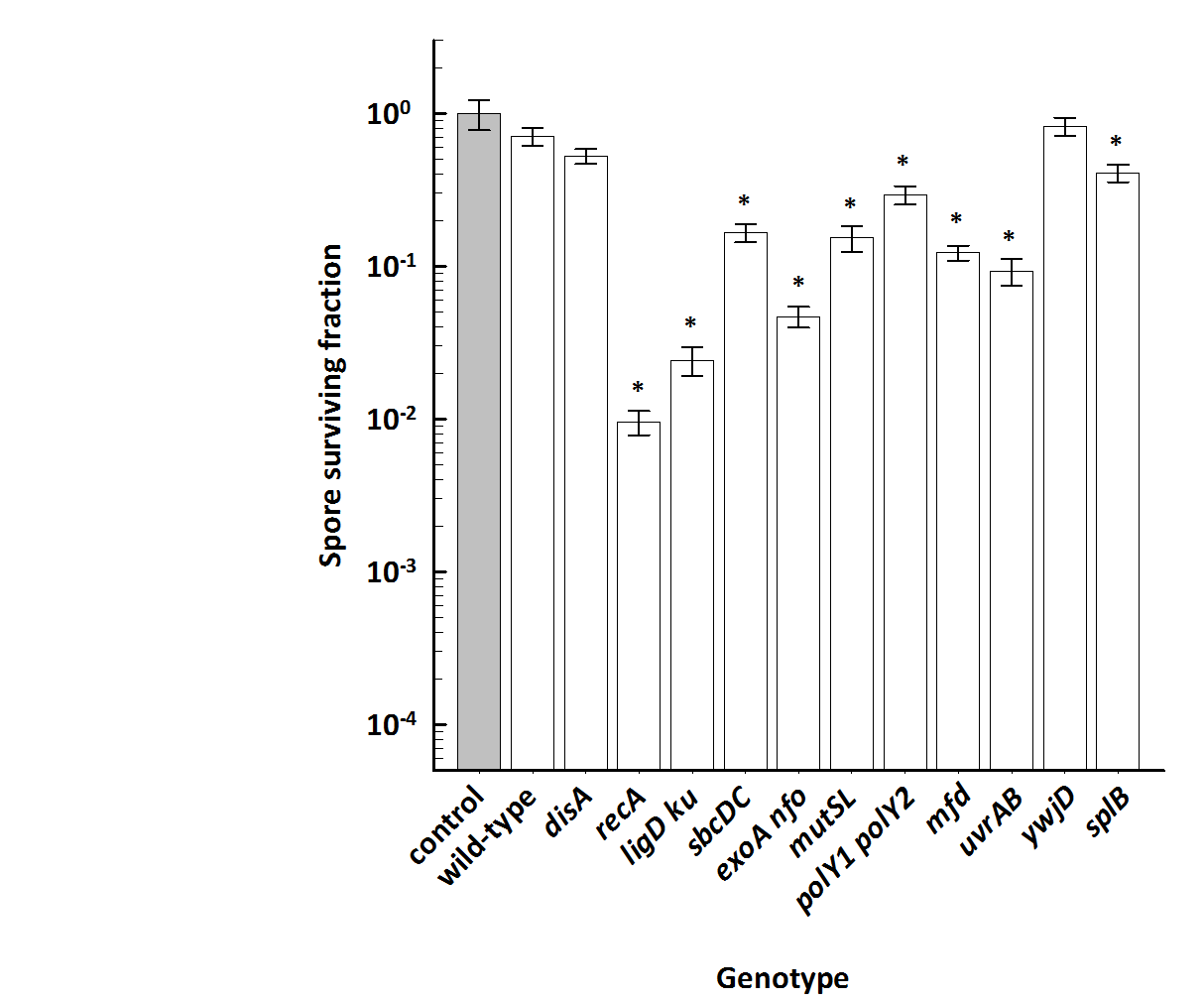

Supplement: Supplementary file 2 [file Data_Sheet_2.ZIP › Supp_9_Cortesao et al_Bacillus subtilis spore resistance to simulated Mars surface conditions.tif]
